# Horseshoes for a Class of Nonuniformly Expanding Random Circle Maps

**DOI:** 10.1007/s00023-025-01555-1

**Published:** 2025-03-05

**Authors:** Jeroen S. W. Lamb, Giuseppe Tenaglia, Dmitry Turaev

**Affiliations:** 1 Department of Mathematics, Imperial College London, London, SW7 2AZ UK; 2 International Research Center for Neurointelligence, The University of Tokyo, Tokyo, 113-0033 Japan; 3 Department of Mathematics and Natural Sciences, Centre for Applied Mathematics and Bioinformatics, Gulf University for Science and Technology, 32093 Halwally, Kuwait

**Keywords:** 37H20, 37B10, 37D25, 60J05

## Abstract

We propose a notion of random horseshoe for one-dimensional random dynamical systems with non-uniform expansion, and we provide sufficient conditions that guarantee their abundant existence, which is shown to hold for a class of non-uniformly expanding random circle endomorphisms with bounded additive noise.

## Introduction and Motivation

One of the most fascinating aspects of dynamical systems is the study of chaos and its properties. In random dynamical systems, the existence of a physical measure [24] with positive top Lyapunov exponent is a popular notion of chaos. Lyapunov exponents are statistical quantities measuring the infinitesimal rate of expansion/contraction along a given direction for typical orbits. If the top one is positive, then the local expansion eventually separates nearby orbits, thus providing a mechanism for causing possibly very complicated dynamics.

In the deterministic setting, a remarkable result by Katok [16] establishes that the dynamics of $\mathcal {C}^{1+\alpha }$ diffeomorphisms preserving an ergodic measure with positive top Lyapunov exponent can be approximated by uniformly hyperbolic invariant sets called horseshoes.

For a diffeomorphism *f* of a manifold *X*, given two boxes $I_0,I_1 \subset X$ and $N \in {\mathbb {N}}$, the set

$$\begin{aligned} H = \bigcap _{j \in {\mathbb {Z}}} f^{-jN}(I_0 \cup I_1) \end{aligned}$$

is called a horseshoe if $f^N\mid _{H}$ is uniformly hyperbolic and is topologically conjugate to the full shift on two symbols, which implies that the bi-infinite sequences of zeros and ones are in one-to-one correspondence with the orbits of points in *H*:

1.1 $$\begin{aligned} \forall \{s_n\}_{n \in {\mathbb {Z}} } \in \{0,1\}^{{\mathbb {Z}}},\,\,\exists \,!\, x \in H :f^{Nj}(x) \in I_{s_j} \qquad \forall j \in {\mathbb {Z}}. \end{aligned}$$

The topological conjugacy implies that $f^N\mid _{H}$ has positive topological entropy and is sensitively dependent on initial conditions. The Katok’s result connects a statistical property, the positivity of the top Lyapunov exponent, to topological dynamical properties.

When aiming to establish a similar correspondence, i.e. between the positivity of the Lyapunov exponent and the existence of horseshoes, for random dynamics, one encounters a number of difficulties. The main issue is to control return times. In deterministic settings, an essential ingredient in Katok’s construction [16] is the identification of two boxes ($I_0,I_1$) with periodic returns. In random systems, however, return times typically depend on noise realizations and may be arbitrarily large with positive probability.

In this paper, we resolve this problem for a class of random perturbations of circle endomorphisms, introduced in Sect. 2. These maps display noise-induced chaos: for small noise amplitude the Lyapunov exponent is negative and typical trajectories synchronize and converge to a random fixed point, but, beyond a critical noise amplitude, the Lyapunov exponent becomes positive. For moderately large noise, we establish both the positivity of Lyapunov exponent and abundance of the horseshoes

Given a set $\Omega$, a map $\theta :\Omega \rightarrow \Omega$ and a family of maps $\{f_{\omega }\}_{\omega \in \Omega }$ with $f_{\omega } \in \mathcal {C}^1({\mathbb {S}}^1)$, consider the iterations

$$\begin{aligned} f^n_{\omega }(x) := f_{\theta ^{n-1}(\omega )} \circ \dots \circ f_{\omega }(x). \end{aligned}$$

In our setting, we endow $\Omega$ with the structure of a probability space, so that the iterations $f^n_{\omega }$ represent a random dynamical system. In Theorem 2.5, which is our main result, we describe a class of random dynamical systems for which we establish an abundance of horseshoes. To be precise, we define the horseshoe as follows:

### Definition 1.1

Let $I_0,I_1$ be two intervals such that $I_0 \cap I_1 = \emptyset$. Let $\omega \in \Omega$ and $\kappa >1$. Then, the pair $(I_0,I_1)$ is an $(\omega ,\kappa )$-horseshoe if there exists a sequence of return times $\{n_k(\omega ) \}_{k \ge 0}$ satisfying

1.2 $$\begin{aligned}  &   \limsup _{k \rightarrow \infty } \frac{n_k(\omega )}{k} < \infty , \end{aligned}$$

1.3 $$\begin{aligned}  &   \quad f^{n_{k+1}(\omega )-n_k(\omega )}_{\theta ^{n_k(\omega )}(\omega )}(I_i) \supseteq I_0 \cup I_1 \qquad \forall i \in \{0,1 \}, \end{aligned}$$

and moreover there exist sets $J(k,\omega )_{i,j} \subset I_i$, which are finite union of intervals, such that

1.4 $$\begin{aligned} f^{n_{k+1}(\omega )-n_k(\omega )}_{\theta ^{n_k(\omega )}(\omega )}(J(k,\omega )_{i,j}) = I_j \end{aligned}$$

and

1.5 $$\begin{aligned} \left| df^{n_{k+1}(\omega )-n_k(\omega )}_{\theta ^{n_k(\omega )}(\omega )}\vert _{J(k,\omega )_{i,j}}\right| >\kappa \qquad \forall k \ge 0. \end{aligned}$$

With this definition, we may consider the set $P(\omega )$, which consists of all points *x* such that $f^{n_k(\omega )}_{\omega }(x) \in I_0 \cup I_1$, for all $k \ge 0$, i.e.

1.6 $$\begin{aligned} P(\omega ):= \{x \in {\mathbb {S}}^1 :f^{n_k(\omega )}_{\omega }(x) \in I_0 \cup I_1\, \forall k \ge 0 \}. \end{aligned}$$

Then, given any sequence of zeros and ones $\alpha =\{ \alpha _k\}_{k \ge 0}$, there is $x \in P(\omega )$ such that $f^{n_k(\omega )}_{\omega }(x) \in I_{\alpha _k}$ for all $k \ge 0$ and this orbit is hyperbolic (we do not claim the 1-to-1 correspondence here, and some orbits in $P(\omega )$ with the same code $\alpha$ might be not hyperbolic). Note that Definition 1.1 is similar to the definition of horseshoe in [13], with the additional hyperbolicity requirement (1.5).

Our main result is exemplified by the following special case:

### Theorem 1.2

For $\frac{1}{2}>\sigma >0$, let ${\mathbb {P}}:= \left( \frac{\textrm{Leb}_{[-\sigma , \sigma ]}}{2\sigma }\right) ^{{\mathbb {N}}}$ be the natural product measure on $\Omega := [-\sigma ,\sigma ]^{{\mathbb {N}}}$ and let $\theta$ denote the shift on $\Omega$, i.e. the map $\theta :\Omega \rightarrow \Omega$ satisfying $\theta (\{\omega _i\}_{i \ge 0}) = \{ \omega _{i+1}\}_{i \ge 0}$. Consider the random dynamical system (RDS) generated by the iterations $f^n_{\omega }:=f_{\theta ^{n-1}(\omega )} \circ \dots \circ f_{\omega }$, where $f_{\omega }$ are circle maps of the form

1.7 $$\begin{aligned} f_{\omega }(x):= L\sin (2\pi (x+\omega _0))+ a \,\,\, (\textrm{mod} \,1), \end{aligned}$$

with $a \in (0,1)$ and $L >0$. Then, there exist constants $\gamma >0$ and $\kappa >1$ such that for every $a \in (0,1)$, $L\ge 9$ and $\sigma \ge \frac{11}{10}L^{-\frac{1}{2}}$

(i) the Lyapunov exponent is positive:
  $$\begin{aligned} \liminf _{n \rightarrow \infty }\frac{1}{n}\log |df^n_{\omega }(x)|>\gamma \log (L) \qquad {\mathbb {P}} \times \textrm{Leb} \,\,\text {a.s.} \,\, (\omega ,x) \in \Omega \times {\mathbb {S}}^1, \end{aligned}$$
(ii) there exists a full ${\mathbb {P}}$-measure set $\tilde{\Omega } \subset \Omega$ such that for every $\omega \in \tilde{\Omega }$ every pair of connected disjoint intervals $(I_0,I_1)$ is an $(\omega ,k)$-horseshoe;
(iii) furthermore, if $L> 25$ and $\sigma > \frac{11}{5\,L^{1/2}}$, the set $P(\omega )$ in (1.6) is hyperbolic and is in one-to-one correspondence with the sequences of zeros and ones (see Remark 2.7).

Theorem 1.2 ensures the existence of horseshoe-like dynamics for a family of non-uniformly expanding RDSs. The choice of the RDSs induced by the family of maps in (1.7) is inspired by [8, 19] who found conditions for positive Lyapunov exponent (as in Theorem 1.2(i)). The novelty of Theorem 1.2 is that it provides a link between the existence of a positive Lyapunov exponent and non-trivial topological dynamics.

Theorem 1.2 is a corollary of our main result Theorem 2.5, in which we prove abundance of random horseshoes for random circle endomorphisms with positive Lyapunov exponents displaying, among other mild conditions, the following key properties:

- fast decay for the tail for the annealed distribution of the Lyapunov exponent and for the recurrences to the critical set (see Hypotheses (H3)–(H4));
- positive probability for intervals of fixed size to grow to full scale (see Hypothesis (H5)).

The proof of Theorem 2.5 employs the theory of hyperbolic times. Hyperbolic times is a standard object in the context of non-uniform hyperbolicity that was introduced in [3] for deterministic systems and extended in [9] to the random case (see Sect. 3 and 4 for further references). Using techniques similar to those developed in [6], we infer from property (*a*) polynomial quenched large-deviation estimates for the Hyperbolic times (Proposition 4.3). This allows us to show that arbitrarily small intervals grow to a fixed size infinitely many times. Then, property (*b*), along with the IID nature of the noise, allows us to show that intervals which grow infinitely often to a fixed size cover eventually the full circle with probability one, thus giving the essential ingredients to conclude the proof of Theorem 2.5.

Theorem 2.5 is a general result that provides abstract conditions for the abundance of horseshoe-like dynamics, which can be applicable in various settings. In particular, Theorem 1.2 provides an example for which the conditions of Theorem 2.5 are actually verifiable. In fact, the same is true for a larger class of random systems which we introduce in Section 2.3 (see Definition 2.8). We call this class $(\sigma ,R)$-*predominantly expanding* RDSs. This is a quantitative generalization of the notion of “predominant expansion” of [8] and contains the RDS generated by the family of maps in (1.7) for $L>>1$ and $\sigma > L^{-1+\varepsilon }$.

This class is composed of random dynamical systems with additive noise, i.e.

$$\begin{aligned} f_{\omega }(x) = f(x+\omega _0), \end{aligned}$$

with $\omega \in \Omega$ as defined in Theorem 1.2, with the following properties:

1. the deterministic map *f* is piecewise $C^2$ and its derivative satisfies $|f'|>R$ in a smooth component of size at least $\frac{1}{R}$;
2. the noise amplitude satisfies
  $$\begin{aligned} \sigma > \frac{1}{R} + \text {Leb}\{ |f'| < R\}. \end{aligned}$$

In Theorem 2.9, we show that $(\sigma ,R)$-predominantly expanding RDSs satisfy the assumptions of Theorem 2.5. In particular, we prove

(i) exponential tail for the annealed distribution of the Lyapunov exponent and for the recurrences to the critical set (see Theorem 7.1 and Theorem 7.2);
(ii) positive probability for intervals of a fixed size to be expanded to cover the full circle (see Proposition 6.4).

The first result is new even in the context of (1.7). A statement similar to (*ii*) can be found, for example, in [7, Lemma 6], where it is proven that the RDS generated by maps of type (1.7) is eventually onto when restricted to intervals of fixed size.

However, our result is applied to a larger class of maps. In particular, in our setting, we only prove the full-scale expansion with positive probability (not with probability one), since we do not require, contrary to [7], a priori upper bound for the size of the contracting region.

As a result, Theorem 2.5 and Theorem 2.9 together ensure that *every pair of disjoint intervals* $(I_0,I_1)$ *is almost surely an* $(\omega ,\kappa )$-*horseshoe for some* $\kappa >1$ for $(\sigma ,R)$-predominantly expanding RDSs.

Earlier examples of horseshoe constructions in random settings were restricted to uniformly hyperbolic RDSs (either mixing on fibres [12], or with an equicontinuous or quasi-periodic base [11]). The existence of non-hyperbolic horseshoe was established for random dynamical systems with positive topological entropy in [13–15].

This paper is organized as follows: In Sect. 2, we recall the basic setup of random dynamical systems, provide sufficient conditions for the existence of a horseshoe and state the main result. We also define the class of predominantly expanding random maps. In Sect. 3, we revisit the theory of hyperbolic times [1] applied to the random setting. In Sect. 4, we obtain estimates on the quenched tail of hyperbolic times. In Sect. 5, we prove the main Theorem 2.5. In sections 6 and 7, we show that the conclusions of the main theorem hold for the class of predominantly expanding random dynamical systems defined in Sect. 2, thus proving Theorem 1.2.

## Statement of the Results

### Hypotheses and Main Result

Given a probability space $(Y,\Sigma ,\nu )$, consider the product space $\Omega := Y^{\otimes {\mathbb {N}}}$, equipped with the product measure ${\mathbb {P}} = \nu ^{\otimes {\mathbb {N}}}$ and the product $\sigma$-algebra $\mathcal {F}$. Let

$$\begin{aligned} \theta :\Omega \rightarrow \Omega , \qquad \theta (\{\omega _i\}_{i \ge 0}) = \{\omega _{i+1}\}_{i \ge 0} \end{aligned}$$

be the shift map acting on $\Omega$. Then, $(\Omega ,\mathcal {F},{\mathbb {P}},\theta )$ is an ergodic dynamical system. Furthermore, let ${\mathbb {S}}^1$ be the unit circle, endowed with the arc-length distance $\textrm{dist}(\cdot ,\cdot )$ and the Borel $\sigma$-algebra $\mathcal {B}({\mathbb {S}}^1)$ induced by this distance. Let also $\textrm{Leb}(\cdot )$ denote the Lebesgue measure on ${\mathbb {S}}^1$. We consider skew product systems of the form

2.1 $$\begin{aligned} \Theta :\Omega \times {\mathbb {S}}^1 \rightarrow \Omega \times {\mathbb {S}}^1, \qquad \Theta (\omega ,x):= (\theta (\omega ),g_{\omega }(x)), \end{aligned}$$

where $\{g_{\omega } \}_{\omega \in \Omega }$ is an $\omega$-measurable family of continuous piecewise $\mathcal {C}^2$ circle endomorphisms such that, for all $\omega \in \Omega$, outside of a finite set $\mathcal {S}_{\omega }$ of non-differentiability points, $g_{\omega }$ is $\mathcal {C}^2$ and its first and second derivatives are uniformly bounded. Furthermore, define the critical set

$$\begin{aligned} \mathcal {C}_{\omega } := \{x \in {\mathbb {S}}^1 \setminus \mathcal {S}_{\omega }\, \,\mid \,\, dg_{\omega }(x)=0 \}, \end{aligned}$$

and let $\mathcal{S}\mathcal{C}_{\omega }:= \mathcal {S}_{\omega } \cup \mathcal {C}_{\omega }$ be the singular set. We say that the families $\{S_{\omega }\}_{\omega \in \Omega }$, $\{\mathcal {C}_{\omega }\}_{\omega \in \Omega }$ and $\{\mathcal{S}\mathcal{C}_{\omega }\}_{\omega \in \Omega }$ are, respectively, the random non-differentiability set, the random critical set and the random singular set for $\{g_{\omega }\}_{\omega \in \Omega }$. The following definition is an adaptation of [1, Definition 4.1].

#### Definition 2.1

Let $\{g_{\omega }\}_{\omega \in \Omega }$ be as above and let $\{\mathcal{S}\mathcal{C}_{\omega }\}_{\omega \in \Omega }$ be its random singular set. We say that $\{\mathcal{S}\mathcal{C}_{\omega }\}_{\omega \in \Omega }$ is ${\mathbb {P}}$ a.s. regular if there exist constants $B>1$ and $\beta > 0$ such that, for almost surely all $\omega \in \Omega$

- for every $$x \in {\mathbb {S}}^1{\setminus } \mathcal{S}\mathcal{C}_{\omega }$$
  $$\begin{aligned} |dg_{\omega }(x)| \ge \frac{1}{B}\textrm{dist}(x,\mathcal{S}\mathcal{C}_{\omega })^{\beta }, \end{aligned}$$
- for every $x,y \in {\mathbb {S}}^1{\setminus } \mathcal{S}\mathcal{C}_{\omega }$ such that $\textrm{dist}(x,y) \le \textrm{dist}(x,\mathcal{S}\mathcal{C}_{\omega })/2$
  $$\begin{aligned} \left| \log \left| dg_{\omega }(x)\right| - \log \left| dg_{\omega }(y)\right| \right| \le B \frac{\textrm{dist}(x,y)}{\textrm{dist}(x,\mathcal{S}\mathcal{C}_{\omega })^{\beta }}. \end{aligned}$$

The setting of this paper concerns a family $\{g_{\omega }\}_{\omega \in \Omega }$ of circle mappings satisfying the following Hypothesis:

#### Hypothesis (H1)

The family $\{g_{\omega }\}_{\omega \in \Omega }$ of circle endomorphisms satisfies:

(i) $g_{\omega }$ depends only on $\omega _0$, and for all $x \in {\mathbb {S}}^1$ the maps $\omega \rightarrow g_{\omega }(x)$ are measurable;
(ii) for ${\mathbb {P}}$ a.s. $\omega \in \Omega$, the maps $x \rightarrow g_{\omega }(x)$ are continuous and piecewise $\mathcal {C}^2$ and their random singular set is regular. Furthermore,
  $$\begin{aligned} {\mathrm{ess\,sup}}_{\omega \in \Omega } ||g_{\omega }||_{\mathcal {C}^2({\mathbb {S}}^1\setminus \mathcal {S}_{\omega })}< \infty . \end{aligned}$$

Given a family $\{g_{\omega } \}_{\omega \in \Omega }$ of circle endomorphisms satisfying Hypothesis (H1), the random composition $g^n_{\omega }$

2.2 $$\begin{aligned} g^n_{\omega }(x)= g_{\theta ^{n-1}(\omega )}(x) \circ \dots \circ g_{\omega }(x), \end{aligned}$$

is a Markov process with associated transition kernel $\{ \mathcal {P}_x(dy):={\mathbb {P}}(g_{\omega }(x) \in dy)\}_{x \in {\mathbb {S}}^1}.$ We recall that $\mu$ is a stationary measure for this Markov process if

$$\begin{aligned} \mu (A) = \int _{{\mathbb {S}}^1}\mathcal {P}_x(A) d\mu (x), \end{aligned}$$

for all Borel sets *A*. Furthermore, $\mu$ is ergodic if and only if the measure ${\mathbb {P}} \otimes \mu$ is ergodic for the skew product $\Theta$ introduced in (2.1). If $\mu$ is ergodic, then by Birkhoff ergodic theorem

$$\begin{aligned} \lim _{n \rightarrow \infty } \frac{1}{n} \sum _{i=0}^{n-1}h(g^i_{\omega }(x)) = \int _{{\mathbb {S}}^1} h(x) d\mu (x) \qquad {\mathbb {P}}\otimes \mu \,\text {a.s}, \end{aligned}$$

for all $h \in L^1(\mu )$. For a more detailed background on Markov processes, see, e.g. [10, 21].

In order to simplify the setting, we introduce the following Hypothesis:

#### Hypothesis (H2)

The Markov process associated with $g^n_{\omega }$ has a unique ergodic stationary measure $\mu$ equivalent to the Lebesgue measure. Furthermore, $(\omega ,x) \mapsto \log |dg_{\omega }(x)| \in L^1\left( \mu \otimes {\mathbb {P}}\right)$.

Note that the ergodic stationary measure $\mu$ is equivalent to Lebesgue if and only if, given any initial condition *x*, the Markov chain $g^n_{\omega }$ starting at *x* can reach every open set with positive probability (cf. also [8, 20]). The ergodicity of $\mu$ and the integrability of the function $\log |dg_{\omega }(x)|$ with respect to ${\mathbb {P}}\otimes \mu$ are required in (H2) to ensure the almost sure existence of the Lyapunov exponent [5]:

#### Definition 2.2

Let $g^n_{\omega }$ be the RDS as in (2.2). The Lyapunov exponent associated with $g^n_{\omega }$ is defined as:

$$\begin{aligned} \lambda _0(\omega ,x):= \liminf _{n \rightarrow \infty } \frac{1}{n} \log \left| \left( dg^n_{\omega }\right) (x) \right| = \liminf _{n \rightarrow \infty } \frac{1}{n} \sum _{i=0}^{n-1} \log |dg_{\theta ^i(\omega )}(g^i_{\omega }(x))|. \end{aligned}$$

The assumption of ergodicity of $\mu$ in (H2) implies that the Lyapunov exponent is ${\mathbb {P}} \otimes \mu$ almost surely constant and equal to

$$\begin{aligned} \lambda _0 = \int _{\Omega }\int _{{\mathbb {S}}^1}\log |\mathrm dg_{\omega }(x)|d\mu (x)d{\mathbb {P}}(\omega ). \end{aligned}$$

We further require that $\lambda _0$ is positive and that finite-time ergodic averages deviate from $\lambda _0$, as stated in the next Hypothesis:

#### Hypothesis (H3)

Let $S_n(\omega ,x):= \sum _{i=0}^{n-1}\log |dg(g^i_{\omega }(x))|$. Then, there exists $\lambda >0$, $\alpha _1 > 0$ and $C_1 \ge 1$ such that, for Lebesgue almost all $x \in {\mathbb {S}}^1$

$$\begin{aligned} {\mathbb {P}}\left( S_n(\omega ,x)< \lambda n\right) \le \frac{C_1}{n^{4+\alpha _1}}, \qquad \forall n \ge 0. \end{aligned}$$

Positivity of $\lambda _0$ follows from combining Hypotheses (H2)-(H3) with a Borel–Cantelli argument. In particular, one has

$$\begin{aligned} \lambda _0 = \lim _{n \rightarrow \infty } \frac{\log |dg^n_{\omega }(x)|}{n} \ge \lambda \qquad {\mathbb {P}}\otimes \mu \,\,\text {a.s.} \end{aligned}$$

We require further assumptions on the behaviour of the orbits, essentially ensuring that typical orbits do not accumulate too fast near the singular set. Given $\delta > 0$ and $\omega \in \Omega$, we define the $\delta$-truncated distance from *x* to the singular set $\mathcal{S}\mathcal{C}_{\omega }$ as

2.3 $$\begin{aligned} \textrm{dist}_{\delta }(x,\mathcal{S}\mathcal{C}_{\omega }):= {\left\{ \begin{array}{ll} 1 \qquad & \text {if}\,\, \textrm{dist}(x,\mathcal{S}\mathcal{C}_{\omega })> \delta , \\ \textrm{dist}(x,\mathcal{S}\mathcal{C}_{\omega }) \qquad & \text {otherwise}. \end{array}\right. } \end{aligned}$$

#### Definition 2.3

We say that $g^n_{\omega }$ exhibits slow recurrence to the random singular set $\{\mathcal{S}\mathcal{C}_{\omega }\}_{\omega \in \Omega }$, if, for every $\varepsilon >0$, there exists a $\delta > 0$ such that, for ${\mathbb {P}} \otimes \mu$ almost all $(\omega ,x) \in \Omega \times {\mathbb {S}}^1$

$$\begin{aligned} \limsup _{n \rightarrow \infty } \frac{1}{n} \sum _{j=0}^{n-1}-\log \textrm{dist}_{\delta }(g^j_{\omega }(x),\mathcal{S}\mathcal{C}_{\theta ^{j}(\omega )})< \varepsilon . \end{aligned}$$

As a consequence of the ergodicity assumption (H2), we have

$$\begin{aligned}&\lim _{n \rightarrow \infty } \frac{1}{n} \sum _{j=0}^{n-1}-\log \textrm{dist}_{\delta }(g^j_{\omega }(x),\mathcal{S}\mathcal{C}_{\theta ^{j}(\omega )})\\&\quad = \int _{\Omega } \int _{{\mathbb {S}}^1}-\log \textrm{dist}_{\delta }(x,\mathcal{S}\mathcal{C}_{\omega }) d\mu (x)d{\mathbb {P}}(\omega ) \qquad {\mathbb {P}} \otimes \mu \,\, \text {a.s.} \end{aligned}$$

In order to control the deviation in the convergence of the above limit, so as to ensure that typical orbits do not get close to the singular set too often, we introduce

#### Hypothesis (H4)

Let $Z_n(\omega ,x,\delta ):= -\sum _{j=0}^{n-1}\log (\textrm{dist}_{\delta }(g^j_{\omega }(x),\mathcal{S}\mathcal{C}_{\theta ^j(\omega )}))$. There exists a function $H(\delta )$ that converges to zero as $\delta \rightarrow 0$ and constants $\alpha _2 >1$ and $C_2 \ge 1$ such that, for Lebesgue almost all $x \in {\mathbb {S}}^1$ and all $\delta >0$ sufficiently small

$$\begin{aligned} {\mathbb {P}}\left( Z_n(\omega ,x,\delta )> H(\delta )n \right) \le \frac{C_2}{n^{4+\alpha _2}}. \end{aligned}$$

Combining Hypotheses (H2) and (H4), it follows that there exists $\tilde{H }(\delta )\le H(\delta )$ such that $\tilde{H}(\delta ) \rightarrow 0$ as $\delta \rightarrow 0$ and

2.4 $$\begin{aligned} \lim _{n \rightarrow \infty } \frac{1}{n} \sum _{j=0}^{n-1}-\log \textrm{dist}_{\delta }(g^j_{\omega }(x),\mathcal{S}\mathcal{C}_{\theta ^j(\omega )}) \le \tilde{H}(\delta ) \qquad {\mathbb {P}}\otimes \mu \,\,\text {a.s.} \end{aligned}$$

In Sect. 7, we show that Hypotheses (H3)–(H4) are satisfied by a class of non-uniformly expanding RDSs with sufficiently large noise amplitude.

In order to state the next Hypothesis, we define the concept of full branch expanding intervals, which will be used in an essential way in the horseshoe construction.

#### Definition 2.4

Let $I \subset {\mathbb {S}}^1$ be an interval. For $\omega \in \Omega$, $\kappa _0>1$ and $K \in {\mathbb {N}}$, we say that *I* is $(\omega ,\kappa _0,K)$-*full branch expanding* if there exists $J \subset I$ and $i \le K$ such that $g^i_{\omega }(J) = {\mathbb {S}}^1$ and $|dg^i_{\omega }\mid _{J}|> \kappa _0$.

In Hypothesis (H5), we require that any interval is $(\omega ,\kappa _0,K)$-full branch expanding with positive probability. This probabilistic assumption on the finite-time growth of intervals, combined with the asymptotic behaviour given in Hypotheses (H3)–(H4), ensures that intervals of any size are eventually onto with full probability, which is used to establish an abundance of random horseshoes.

#### Hypothesis (H5)

There exists $\kappa _0>1$ such that, for all sufficiently small $\eta >0$, there exist a natural number *K* and a real number $\iota \in (0,1)$ such that, for any interval $I \subset {\mathbb {S}}^1$ satisfying $\frac{\eta }{4}\le |I| \le 4\eta$, we have

2.5 $$\begin{aligned} {\mathbb {P}}\left\{ I\, \text {is }(\omega ,\kappa _0,K) \text {-full branch expanding}\right\} > \iota . \end{aligned}$$

In Hypothesis (H6), we additionally require that for ${\mathbb {P}}$ a.s. all $\omega \in \Omega$ there exists an interval $I_{\omega }$ which is full branch and uniformly expanding for $g_{\omega }$. This assumption is relevant for two reasons. First, if $\{g_{\omega }\}_{\omega \in \Omega }$ satisfies (H6), then any $(\omega ,\kappa _0,n)$-full branch expanding interval is also an $(\omega ,\kappa _0,m)$-full expanding branch interval for all $m \ge n$. This is a key property used in the proof of Theorem 2.5. In Sect. 7, we show that (H6) implies (H5) in the case of additive noise with sufficiently large noise amplitude.

#### Hypothesis (H6)

For ${\mathbb {P}}$ almost all $\omega \in \Omega$, there exists an interval $I_{\omega }$ such that $g_{\omega }(I_{\omega }) = {\mathbb {S}}^1$ and

2.6 $$\begin{aligned} \inf _{\omega \in \Omega }|dg_{\omega }|_{I_{\omega }}|>1. \end{aligned}$$

Now we state the main result of this paper.

#### Theorem 2.5

Let $g^n_{\omega }$ be an RDS satisfying (H1)–(H6). Then, there exists a full ${\mathbb {P}}$ measure set $\tilde{\Omega }$ and $\kappa >1$ such that every disjoint pair of intervals $(I_0,I_1)$ is an $(\omega ,\kappa )$-horseshoe for all $\omega \in \tilde{\Omega }$. Furthermore, the random times $\{n_k(\omega )\}_{k \ge 0}$ are measurable and satisfy

$$\begin{aligned} \lim _{k \rightarrow \infty } \frac{n_k(\omega )}{k} = {\mathbb {E}}[n_0]\qquad \forall \omega \in \tilde{\Omega }. \end{aligned}$$

An immediate corollary is the following:

#### Corollary 2.6

Let $g^n_{\omega }$ be an RDS satisfying (H1)–(H6). Then, the system displays sensitive dependence on initial conditions. Furthermore, each of the $(\omega ,\kappa )$-horseshoes carries positive topological entropy greater than or equal to $\frac{\log (2)}{{\mathbb {E}}[n_0]}$.

#### Remark 2.7

Hypotheses (H5)–(H6) can be made stronger in order to ensure that the set $P(\omega )$ in (1.6) is hyperbolic and in one-to-one correspondence with the sequences of zeros and ones. Hypotheses (H5) can be refined as:

#### Hypothesis (H5*)

There exists $\kappa _0>1$ such that, for all sufficiently small $\eta >0$, there exist a natural number *K* and a real number $\iota \in (0,1)$ such that, for any interval $I \subset {\mathbb {S}}^1$ satisfying $\frac{\eta }{4}\le |I| \le 4\eta$, we have

2.7 $$\begin{aligned} {\mathbb {P}}\left\{ I\, \text {is } (\omega ,\kappa _0,K)\text {-double full branch expanding}\right\} > \iota , \end{aligned}$$

where we say that an interval *I* is $(\omega ,\kappa _0,K)$-*double branch expanding* if there exist two adjacent intervals $J_1,J_2 \subset I$ and $i \le K$ such that $g^i_{\omega }(J_1)=g^i_{\omega }(J_2)={\mathbb {S}}^1$ and $|dg^i_{\omega }\mid _{J_1 \cup J_2}|> \kappa _0$. In this sense, we say that, given $J:= J_1 \cup J_2$, $g^i_{\omega }(J)$ “covers the circle twice”.

Hypothesis (H6) can be modified as follows:

#### Hypothesis (H6*)

For ${\mathbb {P}}$ almost all $\omega \in \Omega$, there exists an interval $I_{\omega }$ such that $g_{\omega }(I_{\omega })$ covers the circle twice and

2.8 $$\begin{aligned} \inf _{\omega \in \Omega }|dg_{\omega }|_{I_{\omega }}|>1. \end{aligned}$$

One obtains hyperbolicity of $P(\omega )$ immediately from the fact that, under Hypotheses (H5*)–(H6*), the sets $J(k,\omega )_{i,j}$ in Definition 1.1 are actually connected intervals and the map $g^{n_{k+1}(\omega )-n_k(\omega )}_{\theta ^{n_k(\omega )}(\omega )}$ is monotone on such sets for all $k \ge 0, i,j \in \{ 0,1\}$ (see the proof of this fact in Proposition 5.3).

### A Sketch of the Proof of Theorem 2.5

We proceed to sketch the strategy of the proof of Theorem 2.5, the full details of which are laid out in Sect. 5.

We establish the following fact (see Lemma 5.1):

- There exists $\kappa >1$ and $\alpha >0$ such that, for every interval *I*, the stopping time
  $$\begin{aligned} m(\omega ,I):= \min \{ n \ge 0 \mid \exists J \subset I\,\,\text {such that}\,\, f^n_{\omega }(J) = {\mathbb {S}}^1\,\, \text {and} \,\,|df^n_{\omega }\,\vert _{J}|> \kappa \} \end{aligned}$$
  satisfies
  2.9 $$\begin{aligned} {\mathbb {P}}\left( m(\omega ,I)> l \right) \le \frac{D}{l^{3+\alpha }} \qquad \forall \, l \ge 1, \end{aligned}$$
  for some $D \ge 1$ depending on *I*.

The proof of this fact proceeds as follows:

- First we prove (Proposition 3.2 and Proposition 3.4) that there exists $\kappa _1>1$ and a $\delta _1>0$ small enough such that for every interval $I \subset {\mathbb {S}}^1$ there exists a sequence of almost surely finite stopping times $\tau _k$ and intervals $J_k$ such that
  $$\begin{aligned} |f^{\tau _k}_{\omega }(J_k) |&= 2\delta _1 \\ |df^{\tau _k}_{\omega }\,\vert _{J_k} |&> \kappa _1. \end{aligned}$$
  This is a classical result in the theory of hyperbolic times [2].
- Since $|f^{\tau _k}_{\omega }(J_k)|=2\delta _1$, Hypothesis (H5) implies the existence of $K \in {\mathbb {N}}$, $\kappa _0 >1$ and $\iota \in (0,1)$, both depending on $\delta _1$, such that
  2.10 $$\begin{aligned} {\mathbb {P}}\left( \mathcal {S}'\left( K,f^{\tau _k}_{\omega }(J_k),\kappa _0\right) \right) >i, \end{aligned}$$
  where we denoted
  $$\begin{aligned}&\mathcal {S}'\left( K,f^{\tau _k}_{\omega }(J_k),\kappa _0 \right) \\  &\quad := \left\{ \omega \in \Omega :f^{\tau _k}_{\omega }(J_k)\, \text {is} (\theta ^{\tau _k}(\omega ),\kappa _0,K)\text { -full branch expanding}\right\} . \end{aligned}$$
  Using (2.10) and the independence property of the product measure ${\mathbb {P}}$, we prove Proposition 5.2, which asserts that
  $$\begin{aligned} {\mathbb {P}}\left( \bigcap _{k \ge 0} \mathcal {S}'\left( K,f^{\tau _k}_{\omega }(J_k),\kappa _0\right) ^c\right) =0, \end{aligned}$$
  where $\mathcal {S}'\left( K,f^{\tau _k}_{\omega }(J_k),\kappa _0\right) ^c$ is the complement of the event $\mathcal {S}'\left( K,f^{\tau _k}_{\omega }(J_k),\kappa _0 \right)$ in $\Omega$. As a result, there exists ${\mathbb {P}}$ almost surely a $m_0(\omega )$, an integer $i \le K$ and an interval $J'(\omega ) \subset J_{m_0(\omega )} \subset I$ such that
  $$\begin{aligned} f^{\tau _{m_0}+i}_{\omega }(J')&={\mathbb {S}}^1 \end{aligned}$$
  and
  $$\begin{aligned} |df^{\tau _{m_0}+i}_{\omega }\mid _{J'}| > \kappa := \min \{\kappa _0, \kappa _1\}. \end{aligned}$$
  This proves the claim, as it establishes the almost sure bound $m(\omega ,I)\le \tau _{m_0}+K$ for the stopping time. The estimate in (2.9) is proved in Lemma 5.1.

The bounds in (2.9) are used in Proposition 5.2, assuming Hypothesis (H6), to prove the almost sure existence for any two given intervals $I_0,I_1$ of a sequence of horseshoe return times $\{n_k(\omega ) \}_{k \ge 0}$. The definition of the random sequence $\{n_k(\omega )\}_{k \ge 0}$ implies that the sequences of increments $\{n_{k+1}(\omega )-n_k(\omega ) \}_{k \ge 1}$ and $n_0(\omega )$ are independent and identically distributed. Therefore, Proposition 5.3 establishes positive density (see equation (1.2) in Definition 1.1), using the law of large numbers. Repeating the above construction for all possible pairs of dyadic intervals, we obtain a full ${\mathbb {P}}$-measure set $\tilde{\Omega }$ such that any pair of disjoint intervals is an $(\omega ,\kappa )$-horseshoe. This concludes the proof of Theorem 2.5.

### Application to Random Systems with Predominant Expansion and Large Additive Noise

We proceed to construct the class of RDS to which we apply Theorem 2.5. Consider the probability space $(\Omega ,\mathcal {F},{\mathbb {P}})$, where $\Omega := [-\sigma ,\sigma ]^{{\mathbb {N}}}$, ${\mathbb {P}}:= \left( \frac{\textrm{Leb}\vert _{[-\sigma ,\sigma ]}}{2\sigma }\right) ^{\otimes {\mathbb {N}}}$ and $\mathcal {F}$ is the product Borel $\sigma$-algebra obtained via Kolmogorov extension theorem. Let $f :{\mathbb {S}}^1 \rightarrow {\mathbb {S}}^1$ be a continuous piecewise $\mathcal {C}^2$ map. Then, similarly to what we have done before, we associate to *f* the set $\mathcal {S}$ of non-differentiability points of *f*, the critical set $\mathcal {C}:= \{x \in \mathcal {S}^1 {\setminus } \mathcal {S} :df = 0 \}$ and the singular set $\mathcal{S}\mathcal{C}:= \mathcal {S}\cup \mathcal {C}$.

#### Definition 2.8

Let $\sigma >0$ and $R>1$. We say that a RDS $f^n_{\omega }$ is $(\sigma ,R)$-predominantly expanding if the maps belonging to the family $\{ f_{\omega }\}_{\omega \in \Omega }$ are of the form $f_{\omega } :{\mathbb {S}}^1 \rightarrow {\mathbb {S}}^1$

$$\begin{aligned} f_{\omega }(x):=f(x+\omega _0), \end{aligned}$$

where

1. the generating map *f* satisfies the following conditions:
  (i) *f* is a piecewise $\mathcal {C}^2$ smooth endomorphism with bounded first and second derivatives;
  (ii) Near each point of the singular set $\mathcal{S}\mathcal{C}$ of *f*, either the first or the second derivative of *f* is bounded away from zero.
  (iii) Let $G:= \{x \in {\mathbb {S}}^1 {\setminus } \mathcal {S} \mid |df| > R \}$ be the super-expanding region for *f*. Then *G* is the union of $k \ge 1$ connected components $G_1,\dots ,G_k$ of *G* whose size is larger than $\frac{1}{R}$.
2. The following inequality holds:
  2.11 $$\begin{aligned} \frac{1}{\log (R)}\int _{C}\log | df(x)|dx + \frac{2}{R}+D(R) >0. \end{aligned}$$
  where
  $$\begin{aligned} C:= \{x \in {\mathbb {S}}^1 :|f'(x)| \le 1 \} \end{aligned}$$
  is the contracting region of *f* and
  2.12 $$\begin{aligned} D(R):= \textrm{Leb}\{x \in {\mathbb {S}}^1 :|df| <R \}= \textrm{Leb}\left( {\mathbb {S}}^1{\setminus }G\right) . \end{aligned}$$
3. The noise is sufficiently large, i.e.
  2.13 $$\begin{aligned} \sigma > \frac{1}{R}+D(R). \end{aligned}$$

Note that the $(\sigma ,R)$-predominant expansion is a generalization of the concept of predominant expansion introduced in [8, 20].

Condition (2.13) implies that the noise amplitude is large enough to escape any connected component of ${\mathbb {S}}^1 {\setminus } G$ in one iterate, whilst (2.11) can be interpreted as an estimate for the Lyapunov exponent.

A consequence of (2.13) and (2.11), that is crucial in the proof of Theorem 2.9, is that there exists $h>0$ satisfying

2.14 $$\begin{aligned} \sigma > \frac{D(R)}{2h} \end{aligned}$$

and

2.15 $$\begin{aligned} Z(h):= \log (R)(1-h)+ \frac{1}{2\sigma }\int _C \log |df(x)|dx>0. \end{aligned}$$

As shown in Sect. 7, (2.14) implies that the asymptotic frequency of visits to ${\mathbb {S}}^1{\setminus }G$ is strictly smaller than *h*. Therefore, we can interpret the parameter *h* as an upper bound for the asymptotic frequency of visits to the complement of the super-expanding region *G*.

In order to avoid dealing with the complications arising from the fact that for $\sigma > \frac{1}{2}$ the map $\omega _0 \rightarrow x+\omega _0 \mod 1$ is not one-to-one anymore, we restrict our attention to the “moderate” noise case $\sigma < \frac{1}{2}$. Note that by (2.13) the assumption $\sigma < \frac{1}{2}$ requires also $R>2$.

In such settings, for the class of $(\sigma ,R)$-predominantly expanding random systems, we apply Theorem 2.5 and prove the following result.

#### Theorem 2.9

Any $(\sigma ,R)$-predominantly expanding RDS satisfies all the Hypotheses (H1)–(H6) of Theorem 2.5. As a consequence, there exists $\kappa >1$ such that any pair of disjoint intervals is an $(\omega ,\kappa )$-horseshoe for ${\mathbb {P}}$-almost every $\omega \in \Omega$.

As the RDS generated by the family of maps in (1.7), for any $L \ge 3$, is $(\sigma ,R)$-predominantly expanding for $R=\min \{3,L^{\frac{1}{2}}\}$ and $\sigma$ satisfying (2.13)—for a proof, see Sect. 8—then Theorem 1.2 is a corollary of Theorem 2.9.

## Random Hyperbolic Times

### Definition and Key Property

The aim of this section is to develop the essential tool in our analysis: the hyperbolic times. Hyperbolic times were introduced in [3] for deterministic systems and extended in [9] to the random case. Hyperbolic times have been exploited extensively to construct Young towers [6, 18], to develop thermodynamic formalism [23] and to prove stochastic stability [4]. In this section, we recall the definition and the main properties following the lines of [4]. These properties, along with techniques from [6], are used in Section 4 to obtain a polynomial quenched large-deviation estimates for the tail of hyperbolic times. In order to keep the exposition self-contained, we provide complete proofs.

Let $g^n_{\omega }$ be the RDS induced by (2.2) satisfying (H1)–(H4). Then, there exists $\beta >0$ such that the random critical set of $g^n_{\omega }$ satisfies Definition 2.1. We fix once and for all some positive number $b<\min \left\{ \frac{1}{2},\frac{\beta ^{-1}}{2}\right\}$. The following definition is adapted from [4, Definition 4.1].

#### Definition 3.1

Given $\kappa _1>1$ and $\delta >0$, we say that *n* is a $(\kappa _1,\delta )$-hyperbolic time for $(\omega ,x) \in \Omega \times {\mathbb {S}}^1$ if

$$\begin{aligned} \left| \left( dg^{m}_{\theta ^{n-m}(\omega )}\right) (g^{n-m}_{\omega }(x))\right| \ge \kappa _1^{n-m},\qquad \textrm{dist}_{\delta }(g^{m}_{\omega }(x),\mathcal{S}\mathcal{C}_{\theta ^{m}(\omega )}) \ge \kappa _1^{-b(n-m)} \end{aligned}$$

for all $$0 < m \le n$$.

For $x \in {\mathbb {S}}^1$, let *B*(*x*, *r*) be the open ball around *x* of radius *r*. The following Proposition is an adaptation of [4, Proposition 4.9] and shows that, whenever *n* is a hyperbolic time for $(\omega ,x)$, $g^n_{\omega }$ uniformly expands a sufficiently small neighbourhood of *x* to a neighbourhood of $g^n_{\omega }(x)$ of a fixed radius $\delta _1$. This is the essential property of hyperbolic times needed to prove Theorem 2.5.

#### Proposition 3.2

Suppose *n* is a $(\kappa _1,\delta )$-hyperbolic time for $(\omega ,x)$. Then, for any $\delta _1>0$ small enough, there exists an interval $J \subset B\left( x,\delta _1\kappa _1^{-\frac{n}{2}}\right)$ containing *x* such that $g^n_{\omega }(J)= B(g^n_{\omega }(x),\delta _1)$, $|dg^n_{\omega }\vert _{J}|>\kappa _1^{\frac{n}{2}}$, and $g^i_{\omega }(J) \subset B\left( x,\delta _1\kappa _1^{-\frac{n+i}{2}}\right)$ for all $i= 1,\dots ,n-1$.

#### Proof

First, we state, without proof, a distortion estimate, which follows closely [4, Lemma 4.8]. $\square$

#### Lemma 3.3

Suppose the random singular set is regular. Given $\kappa _1>1$ and $\delta >0$, if *n* is a $(\kappa _1,\delta )$ hyperbolic time for $(\omega ,x)$, then

$$\begin{aligned} |dg_{\omega }(y)| \ge {\kappa _1}^{-\frac{1}{2}}|dg_{\omega }(x)|,\qquad \forall y \in B\left( x,\delta _1\kappa _1^{-\frac{n}{2}}\right) , \end{aligned}$$

for a sufficiently small $\delta _1>0$.

We proceed to prove Proposition 3.2 by induction on *n*. Let $n=1$ be a $(\kappa _1,\delta )$-random hyperbolic time for $(\omega ,x)$. By Lemma 3.3, the ball $B\left( x,\delta _1\kappa _1^{-\frac{1}{2}}\right)$ is expanded by a factor $\left( \kappa _1\right) ^{\frac{1}{2}}$. Therefore, Proposition 3.2 holds for $n=1$. Assuming that the claim holds for *n*, we prove that the claim holds also for $n+1$.

Let $n+1$ be a $(\kappa _1,\delta )$ hyperbolic time for $(\omega ,x)$. Then, *n* is a $(\kappa _1,\delta )$-random hyperbolic time for $(\theta (\omega ),g_{\omega }(x))$. Using the inductive step, there is an interval $I_n \subset B\left( g_{\omega }(x),\delta _1\kappa _1^{\frac{-n}{2}}\right)$ containing $g_{\omega }(x)$ such that

3.1 $$\begin{aligned} g^n_{\theta (\omega )}(I_n) = B(g^{n+1}_{\omega }(x),\delta _1) \end{aligned}$$

and

3.2 $$\begin{aligned} g^i_{\theta (\omega )}(I_n) \subset B\left( x,\delta _1\kappa _1^{\frac{-n+i}{2}}\right) \,\,\forall i = 1,\dots , n-1. \end{aligned}$$

We claim that there is no other interval $I \subset B\left( g_{\omega }(x),\delta _1\kappa _1^{-\frac{n}{2}}\right)$, satisfying (3.1) and (3.2). Indeed, by definition of hyperbolic times, we see that either $\textrm{dist}(g^k_{\omega }(x),\mathcal{S}\mathcal{C}_{\theta _k(\omega )}) \ge \delta > \delta _1$ or $\textrm{dist}(g^k_{\omega }(x),\mathcal{S}\mathcal{C}_{\theta ^k(\omega )})> \kappa _1^{-b(n+1-k)}> \kappa _1^{-\frac{(n+1-k)}{2}}$. In both cases, $\mathcal{S}\mathcal{C}_{\theta ^k(\omega )}$ lies outside of $B\left( g^k_{\omega }(x),\delta _1\kappa _1^{\frac{-n-1+k}{2}}\right)$, for all $k = 1,\dots ,n$. This implies that $g_{\theta ^k(\omega )}$ maps ${B\left( g^k_{\omega }(x),\delta _1\kappa _1^{\frac{-n-1+k}{2}}\right) }$ diffeomorphically onto its image. In particular, by (3.2), $g^{n}_{\theta (\omega )}|_{I_n}$ is injective. Hence, by (3.1), $I=I_n$ and the claim is proved.

Next, we claim that $g_{\omega }\left( B\left( x,\delta _1\kappa _1^{\frac{-n-1}{2}}\right) \right)$ contains $I_n$. We argue by contradiction. Suppose that $g_{\omega }\left( B\left( x,\delta _1\kappa _1^{\frac{-n-1}{2}}\right) \right)$ does not contain $I_n$. Then, at least one of the intervals $g_{\omega }\left( \left[ x-\delta _1\kappa _1^{\frac{-n-1}{2}},x\right] \right)$ and $g_{\omega }\left( \left[ x,x+\delta _1\kappa _1^{\frac{-n-1}{2}}\right] \right)$ is strictly contained in $I_n$. Assume that $g_{\omega }\left( \left[ x-\delta _1\kappa _1^{\frac{-n-1}{2}},x\right] \right) \Subset I_n$ (the other case can be treated analogously). Then, by (3.2),

$$\begin{aligned} g^i_{\omega }\left( \left[ x-\delta _1\kappa _1^{\frac{-n-1}{2}},x\right] \right) \Subset B\left( x,\delta _1\kappa _1^{-\frac{n+i}{2}}\right) \qquad \forall i =1,\dots n \end{aligned}$$

and, by (3.1),

3.3 $$\begin{aligned} g^{n+1}_{\omega }\left( \left[ x-\delta _1\kappa _1^{\frac{-n-1}{2}},x\right] \right) \Subset B(g^{n+1}_{\omega }(x),\delta _1). \end{aligned}$$

Note that, since $n-i$ is a hyperbolic time for $(g^i_{\omega }(x),\theta ^i(\omega ))$, by Lemma 3.3 we have that, for all $i=1,\dots ,n-1$,

$$\begin{aligned} |dg_{\theta ^i(\omega )}(z)| \ge \kappa _1^{-\frac{1}{2}} |dg_{\theta ^i(\omega )}(g^i_{\omega }(x)| \qquad \forall z \in \left[ x-\delta _1\kappa _1^{\frac{-n+i}{2}}g^i_{\omega }(x),g^i_{\omega }(x)\right] . \end{aligned}$$

Furthermore, by (3.2) if $y \in \left[ x-\delta _1\kappa _1^{\frac{-n-1}{2}},x\right]$, then $g^i_{\omega }(y) \in \big [x-\delta _1\kappa _1^{\frac{-n+i}{2}}g^i_{\omega }(x),g^i_{\omega }(x)\big ]$ and we can apply the above inequality for all *i* and $z = g^{i}_{\omega }(y)$ and conclude, by chain rule, that

3.4 $$\begin{aligned} \left| \left( dg^{n+1}_{\omega }\right) (y)\right| = \prod _{i} \left| dg_{\theta ^i(\omega )}(g^i_{\omega }(y))\right| \ge \kappa _1^{-\frac{n+1}{2}}|dg^{n+1}_{\omega }(x)|\ge \kappa _1^{\frac{n+1}{2}}, \end{aligned}$$

for all $y \in \left[ x-\delta _1\kappa _1^{\frac{-n-1}{2}},x\right]$ (recall that $|dg^{n+1}_{\omega }(x)|\ge \kappa _1^{n+1}$ by the definition of the hyperbolic times). As a consequence,

$$\begin{aligned} \left| g^{n+1}_{\omega }\left( \left[ x-\delta _1\kappa _1^{\frac{-n-1}{2}},x\right] \right) \right| \ge \delta _1, \end{aligned}$$

which contradicts (3.3).

One concludes the proof of Proposition 3.2 by choosing the interval

$$\begin{aligned} J := g_{\omega }^{-1}(I_n) \cap \left( B\left( x,\delta _1\kappa _1^{\frac{-n-1}{2}}\right) \right) . \end{aligned}$$

Indeed, $x \in J$ by the injectivity of $g_{\omega }$, the map $g^{n+1}_{\omega }$ takes *J* onto $B(g^{n+1}_{\omega }(x),\delta _1)$ by (3.1), and $\left| dg^{n+1}_{\omega }\vert _{J}\right| > \kappa _1^{\frac{n+1}{2}}$ from computations similar to (3.4). $\square$

### Positive Frequency of Random Hyperbolic Times

The following proposition implies the existence and positive asymptotic frequency for random hyperbolic times. Indeed, we show that for any $(\omega ,x)$, if the orbit at time *N* exhibits a positive finite-time Lyapunov exponent and a sufficiently slow recurrence to the singular set. Equivalently, using the notation in (H3)-(H4), if $S_N(\omega ,x)>\lambda N$ and $Z_N(\omega ,x,\delta )<H(\delta )N$, then the proportion of the hyperbolic times in $\{1,\dots ,N\}$ is bounded away from 0, uniformly for all $(\omega ,x)$ and *n*. This result is used in Section 4 to obtain estimates on the distribution of the hyperbolic times.

The statement and the proof of the following proposition are an adaptation of [4, Proposition 4.4 ].

#### Proposition 3.4

Given $N \in {\mathbb {N}}$, let $S_N(\omega ,x)=\sum _{i=0}^{N-1}\log \left( |dg_{\theta ^i(\omega )}(g^i_{\omega }(x))|\right)$ and ${Z_N(\omega ,x,\delta )=\sum _{i=0}^{N-1}-\log \left( \textrm{dist}_{\delta }(g^i_{\omega },\mathcal{S}\mathcal{C}_{\theta ^i(\omega )})\right) }$. Let $\lambda$ be as in Hypothesis (H3) and $H(\delta )$ as in (H4). Then, there exist $\kappa _1>1$, $\delta >0$ and $\gamma \in (0,1)$, which are independent of $(\omega ,x)$ and *N*, such that, if $S_N(\omega ,x)> \lambda N$ and $Z_N(\omega ,x,\delta )< H(\delta )N$, the number of $\left( \kappa _1,\delta \right)$-hyperbolic times for $(\omega ,x)$ that lie in $\{1,\dots ,N\}$ is larger than $\gamma N$.

The following lemma, due to Pliss, is a key ingredient is the proof of this proposition. For a reference, see [22] or [4, Lemma 4.2].

#### Lemma 3.5

Given $0 < c \le A$ let $\gamma := \frac{c}{A}$. Assume that $a_1,\dots ,a_N$ satisfy $a_j \le A$ for all $1 \le j \le N$ and

$$\begin{aligned} \sum _{j=1}^{N} a_j \ge cN \end{aligned}$$

Then, there exist $l \ge \gamma N$ and $1 \le n_1< \dots < n_l \le N$ such that

$$\begin{aligned} \sum _{j=k}^{n_i} a_j \ge 0 \end{aligned}$$

for every $k= 1,\dots ,n_i$ and $i=1,\dots ,l$.

#### Proof of Proposition 3.4

Consider the sequence

$$\begin{aligned} a_{j+1}:= \log \left| dg_{\theta ^j(\omega )}(g^j_{\omega }(x))\right| - \frac{\lambda }{2}, \qquad j=0,\dots N-1 \end{aligned}$$

Due to (ii) in Hypothesis (H1), $a_j \le A:= {\mathrm{ess\,sup}}_{\omega \in \Omega }||dg_{\omega }||_{\mathcal {C}^1({\mathbb {S}}^1{\setminus } \mathcal {S}_{\omega })}$ for all $j= 1,\dots ,N$. Since $S_N(\omega ,x)>\lambda N$, we have

$$\begin{aligned} \sum _{j=1}^{N} a_j \ge \frac{\lambda }{2}N. \end{aligned}$$

By Lemma 3.5, then there exists a sequence of $l_1 \ge \gamma _1 N$ numbers $1\le n_1,\dots ,n_{l_1}\le N$ such that

$$\begin{aligned} \sum _{j=k}^{n_i}a_j \ge 0, \end{aligned}$$

for all $k = 1,\dots ,n_i$ and $i = 1,\dots ,l_1$, with $\gamma _1:= \frac{\lambda }{2A}$. Using the definition of $a_j$, we may rewrite the above inequality as

3.5 $$\begin{aligned} \left| dg^{n_i-m}_{\theta ^m(\omega )}(g^m_{\omega }(x))\right| \ge e^{\frac{\lambda }{2}(n_i-m)}, \end{aligned}$$

for every $m =0,\dots ,n_i-1$ and $i = 1,\dots ,l$.

Next, we claim that, if $\delta >0$ is small enough, then there exists $\gamma _2 \in (1-\gamma _1,1)$ and $l_2 \ge \gamma _2 N$ numbers $1 \le \tilde{n}_{1},\dots ,\tilde{n}_{l_2}\le N$ satisfying

3.6 $$\begin{aligned} \sum _{j=k}^{\tilde{n}_{i}}\log (\textrm{dist}_{\delta }(g^{j}_{\omega }(x), \mathcal{S}\mathcal{C}_{\theta ^j(\omega )})) \ge - \frac{b\lambda }{8}(\tilde{n}_{i}-k), \end{aligned}$$

for all $k = 1,\dots ,\tilde{n}_i-1$ and $i = 1,\dots ,l_1$, with *b* as in Definition 3.1.

Indeed, since $H(\delta ) \rightarrow 0$ as $\delta \rightarrow 0$, it is possible to choose $\delta >0$ small enough such that $H(\delta ) < \frac{\gamma _1 b \lambda }{8}$. Consider the sequence

3.7 $$\begin{aligned} \tilde{a}_{j+1}:= \log \textrm{dist}_{\delta }\left( g^{j}_{\omega }(x),\mathcal{S}\mathcal{C}_{\theta ^j(\omega )}\right) + \frac{b\lambda }{8},\qquad j=0,\dots , N-1. \end{aligned}$$

As $Z_N(\omega ,x,\delta )\le H(\delta )N$, we have

$$\begin{aligned} \sum _{j=1}^N \tilde{a}_j \ge \left( \frac{b\lambda }{8}- H(\delta )\right) N. \end{aligned}$$

Since $\textrm{dist}_{\delta }(\cdot ,\cdot )\le 1$ by definition, it follows that $\tilde{a}_j \le \frac{b\lambda }{8}$, for all $j=1\dots ,N$. By Lemma 3.5, there exists $l_2 \ge \gamma _2 N$ numbers $1 \le \tilde{n}_1,\dots ,\tilde{n}_{l_2}\le N$ such that

$$\begin{aligned} \sum _{j=k}^{\tilde{n}_i} \tilde{a}_j \ge 0, \end{aligned}$$

for all $k = 1,\dots ,\tilde{n}_i$ and $i=1,\dots ,l_2$, where $\gamma _2= 1- \frac{8H(\delta )}{\lambda }$. From the definition of $\tilde{a}_j$, in (3.7) we obtain (3.6) as claimed.

To finish the proof, note that $\gamma := \gamma _1+\gamma _2-1>0$. Indeed,

$$\begin{aligned} \gamma _1+\gamma _2= \gamma _1 + 1- \frac{8H(\delta )}{\lambda }> \gamma _1 + 1 - \frac{8b\lambda \gamma _1}{8b\lambda } > 1. \end{aligned}$$

Since $\left| \{n_1,\dots ,n_{l_1}\} \right| \ge \gamma _1N$ and $\left| \{\tilde{n}_1,\dots ,\tilde{n}_{l_2}\} \right| \ge \gamma _2N$ and $\gamma := \gamma _1+\gamma _2-1 >0$, the pigeonhole principle implies that the set $\{n_1,\dots ,n_{l_1}\} \cap \{\tilde{n}_1,\dots ,\tilde{n}_{l_2}\}$ contains at least $\gamma N$ elements. By (3.5) and (3.6), every element of this set is a $(e^{\frac{\lambda }{8}},\delta )$-hyperbolic time, which concludes the proof. $\square$

## Quenched Large-Deviation Estimates for Hyperbolic Times

The aim of this section is to prove a quenched large deviation result for hyperbolic times. In order to do it, we exploit the annealed large deviations estimates from Hypotheses (H3)–(H4) along with a Fubini argument taken form [9, Corollary 7.5, Corollary 7.6].

Let $\kappa _1,\delta$ be as in Proposition 3.4. Suppose $\delta _1$ is such that the claim of Proposition 3.2 holds.^1^

### Definition 4.1

Given an interval *I*, we say that *n* is an $(\omega ,I)$-*hyperbolic time* if there exists $x \in I$ such that *n* is a $(\kappa _1,\delta )$-hyperbolic time for $(\omega ,x)$ and $B(x,\delta _1 \kappa _1^{-\frac{n}{2}}) \subset I$.

Let $E_N(\omega )$ be the set of $x \in {\mathbb {S}}^1$ for which the assumptions of Proposition 3.4 fail, i.e. for which we cannot guarantee the density of $(\kappa _1,\delta )$-hyperbolic times in $\{1,\dots ,N\}$ for any $(\omega ,x)$ such that $x \in E_N(\omega )$:

$$\begin{aligned} E_N(\omega )&:= \{x \in {\mathbb {S}}^1 \mid S_N(\omega ,x) \le \lambda N \,\, \text {or}\,\,Z_{N}(\omega ,x,\delta ) \ge H(\delta )N \} \qquad \forall N \ge 0, \end{aligned}$$

where $S_N$ and $Z_N$ are as in Theorem 3.4.

### Proposition 4.2

Let $I \subset {\mathbb {S}}^1$ be an interval. Suppose that *N* is a natural number satisfying $\gamma N > 2\log _{\kappa _1}\left( \frac{4\delta _1}{|I|}\right)$, where $\gamma$ is as in Proposition 3.4. Suppose further that $\omega \in \Omega$ is such that $\textrm{Leb}\left\{ E_N(\omega )\right\} < \frac{|I|}{2}$. Then, in the set $\{1,\dots ,N\}$ there are at least $\gamma N- 2\log _{\kappa _1}\left( \frac{4\delta _1}{|I|}\right)$ $(\omega ,I)$-hyperbolic times.

### Proof

Let *y* be the centre of *I*. If $\textrm{Leb}\left\{ E_N(\omega )\right\} \le \frac{|I|}{2}$, then by Proposition 3.4 there exists at least one $x \in B(y,\frac{|I|}{4})$, which has no fewer than $\gamma N$ $(\kappa _1,\delta )$-hyperbolic times in $\{1,\dots ,N\}$. Suppose that *n* is one of such $(\kappa _1,\delta )$-hyperbolic times, and that

$$\begin{aligned} n > 2\log _{\kappa _1}\left( \frac{4\delta _1}{|I|}\right) . \end{aligned}$$

Then, $B(x,\delta _1\kappa _1^{-\frac{n}{2}}) \subset I$, so that *n* is an $(\omega ,I)$-hyperbolic time. This proves the claim. $\square$

The following Proposition is used extensively in the proof of Theorem 2.5 and establishes an upper bound for the tail of the events $\big \{\omega \in \Omega :\textrm{Leb}(E_N(\omega ))> \frac{|I|}{2} \big \}$.

### Proposition 4.3

For any interval *I*, there exists $C_5 \ge 1$, depending on |*I*|, such that

4.1 $$\begin{aligned} {\mathbb {P}}\left\{ \textrm{Leb}(E_N(\omega )) \ge \frac{|I|}{2} \right\} < C_5 \left( \frac{1}{N}\right) ^{3+\frac{\alpha }{2}} \qquad \forall N \ge 0, \end{aligned}$$

with $\alpha := \max \{\alpha _1,\alpha _2\}$ and $\alpha _1,\alpha _2$ as, respectively, in Hypotheses (H3)–(H4).

### Proof

The computations are similar to [6, Corollary 7.6] and [6, Lemma 7.7], but the exponential tail is replaced by a polynomial tail.

We first show that there exists $C_3 \ge 1$ such that

4.2 $$\begin{aligned} {\mathbb {P}}\left\{ \textrm{Leb}(E_N(\omega ))> \left( \frac{1}{N}\right) ^{\frac{\alpha }{2}} \right\} \le C_3\left( \frac{1}{N}\right) ^{4+\frac{\alpha }{2}} \qquad \forall N \ge 1. \end{aligned}$$

Indeed, suppose the converse is true and there exists $N_0\ge 1$ such that

4.3 $$\begin{aligned} {\mathbb {P}}\left\{ \textrm{Leb}(E_{N_0}(\omega ))> \left( \frac{1}{N_0}\right) ^{\frac{\alpha }{2}} \right\} > C_3\left( \frac{1}{N_0}\right) ^{4+\frac{\alpha }{2}}. \end{aligned}$$

For $x \in {\mathbb {S}}^1$, consider the set of noise realizations

$$\begin{aligned} E_N(x):=\{\omega \in \Omega :S_N(\omega ,x)< \lambda N \,\, \text {or}\,\,Z_{N}(\omega ,x,\delta ) \ge H(\delta )N\}. \end{aligned}$$

By Hypotheses (H3) and (H4), setting $\alpha := \max \{\alpha _1,\alpha _2 \}$, there exists $C_3>1$ such that

$$\begin{aligned} {\mathbb {P}}(E_N(x)) \le \frac{C_3}{N^{4+ \alpha }} \qquad \forall N \ge 0, x \in {\mathbb {S}}^1. \end{aligned}$$

Thus, by Fubini’s theorem,

4.4 $$\begin{aligned} \int _{\Omega } \textrm{Leb}(E_{N_0}(\omega )) d{\mathbb {P}}(\omega )=\int _{{\mathbb {S}}^1}{\mathbb {P}}(E_{N_0}(x))d(x) \le C_3\left( \frac{1}{N_0}\right) ^{4+\alpha }. \end{aligned}$$

However, by (4.3)

$$\begin{aligned} \int _{\Omega } \textrm{Leb}(E_{N_0}(\omega )) d{\mathbb {P}}(\omega )> \left( \frac{1}{N_0}\right) ^{\frac{\alpha }{2}} {\mathbb {P}}\left\{ \textrm{Leb}(E_N(\omega ))> \left( \frac{1}{N_0}\right) ^{\frac{\alpha }{2}}\right\} > C_3\left( \frac{1}{N_0}\right) ^{4+\alpha }, \end{aligned}$$

which contradicts (4.4), and this implies (4.2).

Now, consider

$$\begin{aligned} \tilde{E}_k:= \left\{ \omega \in \Omega :\exists i \ge k\,\text {such that}\, \textrm{Leb}(E_i(\omega )) > \left( \frac{1}{i}\right) ^{\frac{\alpha }{2}}\right\} . \end{aligned}$$

By (4.2), we have

$$\begin{aligned} {\mathbb {P}}(\tilde{E}_k) \le C_3\sum _{i=k}^{\infty } \left( \frac{1}{i}\right) ^{4+\frac{\alpha }{2}} \le C_4\left( \frac{1}{k}\right) ^{3+\frac{\alpha }{2}} \qquad \forall k \in {\mathbb {N}}, \end{aligned}$$

for some constant $C_4 \ge 1$. Since $\lim _{n \rightarrow \infty } {\mathbb {P}}(\tilde{E}_n)=0$, the random time

$$\begin{aligned} N_1(\omega ):= \min \{n \ge 1 \mid \omega \notin \tilde{E}_n \} \end{aligned}$$

is finite almost surely. By the definition of $\tilde{E}_{N_1(\omega )}$, for all $N \ge N_1(\omega )$

4.5 $$\begin{aligned} \textrm{Leb}(E_N(\omega )) \le \left( \frac{1}{N}\right) ^{\frac{\alpha }{2}}. \end{aligned}$$

Furthermore,

4.6 $$\begin{aligned} {\mathbb {P}}\{ N_1(\omega ) > k\} \le C_4\left( \frac{1}{k}\right) ^{3+\frac{\alpha }{2}}. \end{aligned}$$

Finally, for $k_0$ sufficiently large so that $\left( \frac{1}{k_0}\right) ^{\frac{\alpha }{2}} \le \frac{|I|}{2}$, by (4.5), if $\textrm{Leb}(E_N(\omega )) \ge \frac{|I|}{2}$, then $N_1(\omega )>N-k_0$ and (4.1) follows from (4.6). $\square$

## Random Horseshoe

In this section, we prove Theorem 2.5. The main ingredients in the proof of this theorem are Lemma 5.1, Proposition 5.2 and Proposition 5.3.

Let $\kappa _0$ be as in Hypothesis (H5) and $\kappa _1$ as in Proposition 3.4, take $\kappa := \min \{\kappa _0,\kappa _1\}$. Let *I* be any given interval and consider the following random time

5.1 $$\begin{aligned} m(\omega ,I):= \min \{n \ge 0 \mid I \,\,\text {is an}\,\,(\omega ,n,\kappa )\text {-full expanding branch}\}. \end{aligned}$$

In the following Lemma we show that the random time *m* is almost surely finite with at least a power-law tail.

### Lemma 5.1

For every interval *I*, there exists a constant $C_7 \ge 1$ such that

5.2 $$\begin{aligned} {\mathbb {P}}\{ m(\omega ,I)> l \} \le C_7 \left( \frac{1}{l}\right) ^{3+\frac{\alpha }{4}} \qquad \forall l \ge 0. \end{aligned}$$

In particular, $m(\omega ,I)$ is almost surely finite. Furthermore, if we assume (H5*) instead of (H5) the result is the same with

$$\begin{aligned} m(\omega ,I):= \min \{n \ge 0 \mid I \,\,\text {is an}\,\,(\omega ,n,\kappa )\text {-double full expanding branch}\}. \end{aligned}$$

We defer the proof of this Lemma to the end of the section, as it is involved.

Define recursively the random sequence

$$\begin{aligned} n_0(\omega ):= 0 \qquad \forall \omega \in \Omega , \end{aligned}$$

and

5.3 $$\begin{aligned} n_{k}(\omega ):= \max \{m(\theta ^{n_{k-1}(\omega )}(\omega ),I_0),m(\theta ^{n_{k-1}(\omega )}(\omega ),I_1) \}, \qquad \forall \omega \in \Omega .\nonumber \\ \end{aligned}$$

By Lemma 5.1, $n_k$ is almost surely finite for all $k \ge 0$. In the following Proposition, we show how to use the definition of $\{n_k(\omega )\}_{k \ge 0}$ to construct symbolic dynamics between $I_0$ and $I_1$.

### Proposition 5.2

Given two disjoint intervals $I_0,I_1 \subset {\mathbb {S}}^1$, there exists a full measure set $\hat{\Omega }$, such that for all $\omega \in \hat{\Omega }$, $i,j \in \{0,1\}$ and $k \ge 0$, there exists a set $J^{i,j}_k(\omega ) \subset I_i$ such that

$$\begin{aligned} g^{n_{k+1}(\omega )-n_k(\omega )}_{\theta ^{n_k(\omega )}(\omega )}(J^{i,j}_k(\omega ))&= I_j,\\ \left| dg^{n_{k+1}(\omega )-n_k(\omega )}_{\theta ^{n_{k}(\omega )}(\omega )}|_{J^{i,j}_k(\omega )}\right|&> \kappa > 1. \end{aligned}$$

### Proof

Let $\hat{\Omega }$ be such that $n_k$ is finite for all $\omega \in \hat{\Omega }$ and all $k \ge 0$. Consider the case $k= 0$. Without loss of generality, let $m(\omega ,I_0) > m(\omega ,I_1)$. Let $E_{\omega }:= \{x \in {\mathbb {S}}^1 \mid |dg_{\omega }|> 1\}$. By Hypothesis (H6), up to restricting to a full measure subset of noise realizations, we can assume that $g_{\theta ^s(\omega )}(E_{\theta ^s(\omega )})= {\mathbb {S}}^1$ for all $\omega \in \hat{\Omega }$ and all $s \ge 0$. As a consequence, if $\omega \in \hat{\Omega }$ there are sets (which are intervals if we apply Hypothesis (H6*)) $E'_{{\omega },j} \subset E_{\theta ^{m(\omega ,I_1)}(\omega )}$, $j \in \{0,1\}$, such that $g^{q}_{\theta ^{m(\omega ,I_1)}(\omega )} \subset E_{\theta ^{m(\omega ,I_1)+q}(\omega )}$ for every $q \le m(\omega ,I_0)-m(\omega ,I_1)$ and

5.4 $$\begin{aligned} g^{m(\omega ,I_0)-m(\omega ,I_1)}_{\theta ^{m(\omega ,I_1)}(\omega )}(E'_{{\omega },j})= I_j. \end{aligned}$$

By definition of $m(\omega ,I)$, we can find the sets $\{J^{i,j}_0(\omega )\}_{i,j \in \{0,1\}}$ such that $J^{i,j}_0(\omega ) \subset I_i$ and

$$\begin{aligned} g^{m(\omega ,I_1)}_{\omega }(J^{1,j}_0(\omega ))&= E'_{{\omega },j},\\ g^{m(\omega ,I_0)}_{\omega }(J^{0,j}_0(\omega ))&= I_j, \\ \left| dg^{m(\omega ,I_i)}_{\omega }\vert _{J^{i,j}_{0}(\omega )}\right|&> \kappa . \end{aligned}$$

By (5.4)

$$\begin{aligned} g^{m(\omega ,I_0)}_{\omega }(J^{1,j}_0(\omega ))&= g^{m(\omega ,I_0)-m(\omega ,I_1)}_{\theta ^{m(\omega ,I_1)}(\omega )} \left( g^{m(\omega ,I_1)}_{\omega }(J^{1,j}_0(\omega ))\right) \\&= g^{m(\omega ,I_1)-m(\omega ,I_0)}_{\theta ^{m(\omega ,I_1)}(\omega )}(E'_{\omega ,j})= I_j. \end{aligned}$$

This concludes the proof for $k=0$ because we assumed that $n_0(\omega )=m(\omega ,I_0)$. For the case $k \ge 1$, the proof is the same up to replacing $\omega$ with $\theta ^{n_{k-1}(\omega )}(\omega )$. $\square$

In the following Proposition, we establish positive density for the sequence $\{n_k(\omega )\}_{k \ge 0}$.

### Proposition 5.3

For every pair of disjoint intervals $I_0,I_1 \subset {\mathbb {S}}^1$, there exists a full measure set $\overline{\Omega } \subset \hat{\Omega }$ such that

$$\begin{aligned} \lim _{k \rightarrow \infty }\frac{n_k(\omega )}{k}= E[n_0(\omega )] \qquad \forall \omega \in \overline{\Omega }. \end{aligned}$$

### Proof

Observe that

$$\begin{aligned} n_k= n_0+ (n_1-n_0)+ \dots + (n_{k}-n_{k-1}). \end{aligned}$$

The random variables $\{n_{j+1}-n_j\}_{j \ge 0}$ and $n_0$ are independent and identically distributed. Furthermore, by (5.2)

$$\begin{aligned} {\mathbb {E}}[(n_0)^2]&\le \sum _{l \ge 0} l^2 {\mathbb {P}}(n_0(\omega ) > l-1) \\&\le C_6 \sum _{l \ge 0}l^2 \left( \frac{1}{l}\right) ^{3+ \frac{\alpha }{4}}\\&\le C_6\sum _l \frac{1}{l^{1+\frac{\alpha }{4}}} < \infty . \end{aligned}$$

By the law of large numbers, we conclude that

$$\begin{aligned} \lim _{k \rightarrow \infty }\frac{n_k(\omega )}{k}= {\mathbb {E}}[n_0(\omega )], \qquad {\mathbb {P}} \,\,\text {a.s.} \end{aligned}$$

$$\square $$

### Proof of Theorem 2.5

Fix two disjoint intervals $I_0,I_1 \subset {\mathbb {S}}^1$ and consider the random sequence $\{n_k(\omega )\}_{k \ge 0}$. By Proposition 5.2 and 5.3, there is a full ${\mathbb {P}}$-measure set $\overline{\Omega }(I_0,I_1)$ such that $\{n_k(\omega )\}_{k \ge 0}$ satisfies (1.2), (1.3),(1.4) and (1.5) for all $\omega \in \overline{\Omega }(I_0,I_1)$. By Definition 1.1, the pair $(I_0,I_1)$ is an $(\omega ,\kappa )$-horseshoe for all $\omega \in \overline{\Omega }(I_0,I_1)$. This implies the theorem with the full measure set

$$\begin{aligned} \tilde{\Omega }:= \bigcap _{I_0,I_1 \,\text {dyadic intervals}} \overline{\Omega }(I_0,I_1). \end{aligned}$$

$$\square $$

The rest of this section is dedicated to the proof of Lemma 5.1.

### Proof of Lemma 5.1

Fix an interval $I \subset {\mathbb {S}}^1$. Let $\delta _1>0$ be small enough such that Hypothesis (H5) ensures, for $\eta = \delta _1$, the existence of $\kappa _0>1$, $K \in {\mathbb {N}}$ and $\iota >0$ satisfying (2.5). Define the sequence of *K*-sparse hyperbolic times

$$\begin{aligned} \tau _1(\omega )&:= \min \{ n \ge 1 \mid n \text {is a} (\omega ,I) \text {-hyperbolic time } \},\\ \tau _{k+1}(\omega )&:=\min \{n > \tau _k(\omega )+K \mid n \text {is a } (\omega ,I)\text {-hyperbolic time} \}, \end{aligned}$$

where the $(\omega ,I)$-hyperbolic times are as in Definition 4.1. Let $k_l(\omega ):= \max \{ i \mid \tau _i(\omega ) \le l\}$, i.e. $k_l(\omega )$ is the number of *K*-sparse hyperbolic times that lie in $\{1,\dots ,l\}$. Before proceeding with the proof, we establish an estimate which will be used in the conclusion of this section. $\square$

### Proposition 5.4

Let $\gamma \in (0,1)$ be as in Proposition 3.4. Let *s* be a natural number such that $(K+1) s \le \gamma l- 2\log _{\kappa }\left( \frac{4\delta _1}{|I|}\right)$ if $l \le \frac{2\log _{\kappa }\left( \frac{4\delta _1}{|I|}\right) }{\gamma }$; otherwise $s=0$. Then,

$$\begin{aligned} {\mathbb {P}}(k_l(\omega ) = s) \le C_6 \left( \frac{1}{l}\right) ^{3+\frac{\alpha }{2}}. \end{aligned}$$

### Proof

Let $t_l(\omega )$ be the number of $(\omega ,I)$-hyperbolic times less or equal to *l*. It is readily verified that

5.5 $$\begin{aligned} k_l(\omega ) \ge \frac{1}{K+1}t_l(\omega ), \qquad \forall l \ge 0. \end{aligned}$$

By Proposition 4.2, if $s(K+1) \le \max \left\{ \gamma l - 2\log _{\kappa }\left( \frac{4\delta _1}{|I|}\right) ,0 \right\}$, then either $l \le \frac{2\log _{\kappa }\left( \frac{4\delta _1}{|I|}\right) }{\gamma }$ or

$$\begin{aligned} \left\{ k_l(\omega ) \le s \right\} \subset \left\{ \textrm{Leb}(E_l(\omega )) \ge \frac{|I|}{2} \right\} , \end{aligned}$$

and the result follows immediately from Proposition 4.3 with $l=N$. $\square$

By definition of the $(\omega ,I)$-hyperbolic times, the image $f^{\tau _j}_{\omega }(I)$ contains a ball of radius $\delta _1$ for all $j \in {\mathbb {N}}$. Let $H(\omega ,j)$ be such ball. Define the set of noise realizations

$$\begin{aligned} \mathcal {S}\left( K,j,\kappa \right) := \{ H(\omega ,j) \,\text {is not } (\theta ^{\tau _j}(\omega ),K,\kappa )\text {- full branch expanding}\},\qquad j \ge 0. \end{aligned}$$

Observe that if $m(\omega ,I)>l$, then *I* is not $(\omega ,l,\kappa )$-full branch expanding. This implies that $H(\omega ,j)$ is not $(\theta ^{\tau _j}(\omega ),K,\kappa )$-full branch expanding, for all $j = 1,\dots , k_l(\omega )-1$. Indeed, if for some $j_0$ the set $H(\omega ,j_0)$ were $(\theta ^{\tau _{j_0}}(\omega ),K,\kappa )$-full branch expanding, then the interval

$$\begin{aligned} V(\omega ,j_0):= \left( f^{\tau _{j_0}}_{\omega }\right) ^{-1}(H(\omega ,j_0)) \cap I \subset I \end{aligned}$$

would satisfy, for some $i \le K$, that $f^{\tau _{j_0}+i}_{\omega }(V(\omega ,j_0))= {\mathbb {S}}^1$ and $|(df^{\tau _{j_0}+i}_{\omega })_{\mid V(\omega ,j_0)}|>\kappa$, which would, in turn, imply that *I* is $(\omega ,l,\kappa )$-full branch expanding. Therefore, we can write

$$\begin{aligned} {\mathbb {P}}\left( m(\omega ,I)> l\right)&=\sum _{s=0}^l{\mathbb {P}}\left( m(\omega ,I) > l\,\,\text {and}\,\,k_l(\omega )=s\right) \\  &\le \sum _{s=0}^l{\mathbb {P}}\left( \bigcap _{j=1}^{k_l(\omega )-1} \mathcal {S}\left( K,j ,\kappa \right) \,\,\text {and}\,\, k_l(\omega )=s \right) \\&= \sum _{s=0}^l {\mathbb {P}}\left( \bigcap _{j=1}^{s-1} \mathcal {S}\left( K,j,\kappa \right) \,\,\text {and}\,\, k_l(\omega )=s \right) . \end{aligned}$$

Therefore, by Hölder inequality, we have

5.6 $$\begin{aligned} {\mathbb {P}}\left( m(\omega ,I)>l \right) \le \sum _{s=0}^l \left( {{\mathbb {P}} \left( \bigcap _{j=1}^{s-1} \mathcal {S}\left( K,j,\kappa \right) \right) }\right) ^{\frac{\alpha }{12+2\alpha }} \left( {{\mathbb {P}}(k_l=s)}\right) ^{\frac{12+\alpha }{12+2\alpha }},\nonumber \\ \end{aligned}$$

where $\alpha$ is as in Proposition 4.3.

Let $\iota$ be as in Hypothesis (H5). We claim that

5.7 $$\begin{aligned} {\mathbb {P}}\left( \bigcap _{j=1}^{s-1} \mathcal {S}\left( K,j,\kappa \right) \right) \le (1-\iota )^{s-1}. \end{aligned}$$

Let $$V_i= \bigcap _{j=1}^i\mathcal {S}\left( K,j,\kappa \right) $$, then

5.8 $$\begin{aligned} {\mathbb {P}}\left( V_{s-1}\right) ={\mathbb {E}}\left[ {\mathbb {P}} \left( \mathcal {S}\left( K,s-1,\kappa \right) |\mathcal {F}_{\tau _{s-1}}\right) (\omega )\cdot 1_{V_{s-2}}(\omega )\right] , \end{aligned}$$

where $\mathcal {F}_{\tau _{s}}$ denotes the past $\sigma$-algebra up to time $\tau _{s}$, i.e

$$\begin{aligned} \mathcal {F}_{\tau _{s}} := \left\{ A \in \mathcal {F} :A \cap \{ \tau _s \le n \} \in \mathcal {F}_n\;\;\forall n \ge 0 \right\} , \end{aligned}$$

and

$$\begin{aligned} \mathcal {F}_{n} = \sigma (\omega _0,\dots ,\omega _{n-1}) \end{aligned}$$

denotes the sigma-algebra generated by projecting $\Omega$ into the first *n* coordinates. Since the length of $H(\omega ,s-1)$ is equal to $2\delta _1 \in (\frac{\eta }{4},4\eta )$, then by Hypothesis (H5),

$$\begin{aligned} {\mathbb {P}}\left( \mathcal {S}\left( K,s-1,\kappa \right) | \mathcal {F}_{\tau _{s-1}}\right) (\omega ) \le 1-\iota . \end{aligned}$$

Therefore, by (5.8)

$$\begin{aligned} {\mathbb {P}}\left( \bigcap _{j=1}^{s-1}\mathcal {S}\left( K,j,\kappa \right) \right) \le (1-\iota ){\mathbb {P}}\left( \bigcap _{j=1}^{s-2}\mathcal {S}\left( K,j,\kappa \right) \right) , \end{aligned}$$

and inequality (5.7) follows by induction.

It follows from equations (5.6)–(5.7) and Proposition 5.4 that

$$\begin{aligned}&{\mathbb {P}}(m(\omega ,I)>l) \le (1-\iota )^{-\frac{\alpha }{12+2\alpha }}\sum _{s = 0}^l (1-\iota )^{s\frac{\alpha }{12+2\alpha }} {{\mathbb {P}}(k_l(\omega )=s)}^{\frac{12+\alpha }{12+2\alpha }}\\  &\le (1-\iota )^{-\frac{\alpha }{12+2\alpha }}C_6\left( \frac{1}{l}\right) ^{\left( 3+\frac{\alpha }{2}\right) (\frac{12+\alpha }{12+2\alpha })}\sum _{s(K+1) \le \max \left\{ 0,\gamma l- 2\log _{\kappa }\left( \frac{4\delta _1}{|I|}\right) \right\} } (1-\iota )^{s\frac{\alpha }{12+2\alpha }}\\&\qquad +(1-\iota )^{-\frac{\alpha }{12+2\alpha }}\sum _{s(K+1) > \max \left\{ 0,\gamma l- 2\log _{\kappa }\left( \frac{4\delta _1}{|I|}\right) \right\} } (1-\iota )^{s\frac{\alpha }{12+2\alpha }} \\&\quad \le C_6\left( \frac{1}{l}\right) ^{3+\frac{\alpha }{4}}(1-\iota )^{-\frac{\alpha }{12+2\alpha }}\left( \sum _{s \ge 0} (1-\iota )^{s\frac{\alpha }{12+2\alpha }}\right) \\&\qquad + \left( (1-\iota )^{\frac{-\alpha \log \left( \frac{4\delta _1}{|I|}\right) }{12+2\alpha }}\right) \left( (1-\iota )^{\frac{\alpha }{12+2\alpha } \frac{\gamma }{K+1}}\right) ^l\left( \sum _{s \ge 0} (1-\iota )^{s\frac{\alpha }{12+2\alpha }}\right) \\&\quad \le C_7\left( \frac{1}{l}\right) ^{3+\frac{\alpha }{4}}, \end{aligned}$$

for some constant $C_7 \ge 1$, which concludes the proof of Lemma 5.1. $\square$

## A Random Horseshoe for Predominantly Expanding RDS: Lyapunov Exponent and Invariant Measure

Sections 6 and 7 are devoted to the proof of Theorem 2.9. In particular, we need to verify that any $(\sigma ,R)$-predominantly expanding RDS (see Definition 2.8) satisfies Hypotheses (H1)–(H6). In Section 6, we check that any $(\sigma ,R)$-predominantly expanding RDS satisfies Hypotheses (H1)–(H2) and (H5)–(H6), whilst in Section 7 we focus on the remaining Hypotheses (H3)–(H4).

From now on, using the same notation as in Section 6, we set $\Omega := [-\sigma ,\sigma ]^{{\mathbb {N}}}$, ${\mathbb {P}}:= \left( \frac{\textrm{Leb}\vert _{[-\sigma ,\sigma ]}}{2\sigma }\right) ^{\otimes {\mathbb {N}}}$ given the noise amplitude $\sigma >0$ and we let $\mathcal {F}$ be the product Borel $\sigma$-algebra on $\Omega$ obtained via Kolmogorov extension theorem.

We remind—see Section 2.3 for further details—that a $(\sigma ,R)$-predominantly expanding RDS $f^n_{\omega }$ is generated by a family of iterations $\{f_{\omega }\}_{\omega \in \Omega }$ that has a regular random singular set $\{\mathcal{S}\mathcal{C}_{\omega }\}_{\omega \in \Omega }$ (see Definition 2.1) and satisfies

$$\begin{aligned} f_{\omega }(x):= f(x + \omega _0), \end{aligned}$$

for a given function *f* that satisfies the requirements in Definition 2.8. Due to the behaviour of the first and the second derivative of *f* around the singular set $\mathcal {S}$, item (*ii*) of Definition 2.8, one can prove that $\mathcal{S}\mathcal{C}$ is regular (see Definition 2.1). Recall that $\mathcal{S}\mathcal{C}$ is the union of the non-differentiability set $\mathcal {S}$ and of the critical set $\mathcal {C}$. Furthermore, recall that $G= \{|df|>R \}$ is the super-expanding set, and there are $G_1,\dots ,G_k$ connected components of *G* that satisfy $|G_i| \ge \frac{1}{R}$.

The following proposition shows a crucial property of $(\sigma ,R)$-predominantly expanding maps, which is frequently used in this section, i.e. that there exists a $\Delta$ in the super-expanding region such that $|\Delta |\le \frac{1}{R}$, $f(\Delta )= {\mathbb {S}}^1$ and $\Delta$ satisfies an accessibility condition (see equation (6.4)).

### Proposition 6.1

Let $f^n_{\omega }$ be $(\sigma ,R)$-predominantly expanding. Let $G_1$ be one of the connected components of *G* in item (*iii*) of Definition 2.8 and let *c* be its middle point. Let

6.1 $$\begin{aligned} \Delta := \left[ c-\frac{1}{2R},c+\frac{1}{2R}\right] . \end{aligned}$$

Then, $$\Delta \subset G_1$$ and $$f(\Delta )= {\mathbb {S}}^1$$.

Furthermore, given $\gamma >0$, consider the sets

6.2 $$\begin{aligned} \begin{array}{ll} \Delta (\gamma ) & := \{ x \in \Delta \mid d\left( x,{\mathbb {S}}^1 {\setminus }G\right)>\gamma \}, \\ E(\gamma ) & := \{ x \in E \mid d\left( x,{\mathbb {S}}^1{\setminus }E\right) >\gamma \}, \end{array} \end{aligned}$$

where *E* is the expanding region for *f*, i.e.

6.3 $$\begin{aligned} E:= \left\{ x \in {\mathbb {S}}^1 :|f'(x)|>1 \right\} . \end{aligned}$$

Then, there exists a $\gamma >0$ and $q \in (0,1)$ such that

6.4 $$\begin{aligned} {\mathbb {P}}\left( x+\omega _0 \in E(\gamma )\, \,\,\text {and}\,\, f_{\omega }(x) \in \Delta (\gamma )\right) > q, \end{aligned}$$

for all $$x \in {\mathbb {S}}^1$$.

### Proof

The first assertion of this proposition follows from the fact that any interval inside $G_1$ of size at least $\frac{1}{R}$ is mapped into the full circle due to Lagrange theorem. For the proof of (6.4), observe that, due to (2.12), for any $x \in {\mathbb {S}}^1$ there exists $G_{i}$ such that $d(x,G_i) \le \frac{D(R)}{2}$. Using (2.13) and the fact that $f(G_i)= {\mathbb {S}}^1$, choosing $\gamma$ small enough such that

$$\begin{aligned} \left( G_i \cap \Delta (\gamma )\right) \cap f^{-1}(\Delta (\gamma )) \ne \emptyset \,\, \forall i =1,\dots , k, \end{aligned}$$

we conclude that

$$\begin{aligned} \inf _{x \in {\mathbb {S}}^1}{\mathbb {P}} \left( x+\omega _0 \in f^{-1}(\Delta (\gamma ))\cap G_i \right) >0, \end{aligned}$$

from which (6.4) follows immediately. $\square$

### Regularity of the Singular Set and Uniqueness of Ergodic Stationary Measure

The main result of this subsection is the following proposition:

#### Proposition 6.2

Let $f^n_{\omega }$ be any $(\sigma ,R)$-predominantly expanding random dynamical system. Then, (H1) and (H2) are satisfied. In particular, the RDS $f^n_{\omega }$ has an absolutely continuous unique ergodic stationary measure $\mu$ such that $\log |df_{\omega }(x)| \in L^1({\mathbb {P}} \otimes \mu )$.

#### Proof

The proof that (H1) is verified is an easy consequence of item (*i*) in Definition 2.8 and is left to the reader.

To prove existence and uniqueness of the ergodic measure, due to compactness of ${\mathbb {S}}^1$, it is sufficient to prove that any initial condition $x \in {\mathbb {S}}^1$ can reach any open set of the circle with positive probability ( see [17, chapter 1]). For a $(\sigma ,R)$-predominantly expanding RDS $f^n_{\omega }$, this fact is established in the following Lemma, which uses the existence of $\Delta$ as in (6.1) and the sufficiently large noise amplitude. $\square$

#### Lemma 6.3

For any $x \in {\mathbb {S}}^1$ and for any non-empty open set $A \subset {\mathbb {S}}^1$, ${\mathbb {P}}\{f_{\omega }^2(x) \in A\}>0$.

#### Proof

Fix $x \in {\mathbb {S}}^1$ and take a non-empty open set $A \subset {\mathbb {S}}^1$. By (6.4),

6.5 $$\begin{aligned} {\mathbb {P}}\left\{ f_{\omega }(x) \in \Delta \right\} >0. \end{aligned}$$

Since $$f(\Delta ) = {\mathbb {S}}^1$$, $$f^{-1}(A) \cap \Delta \ne \emptyset $$. Then,

$$\begin{aligned} {\mathbb {P}}\left\{ f^{2}_{\omega }(x) \in A\right\}&\ge {\mathbb {P}}\left\{ f_{\omega }(x) +\omega _{1} \in \Delta \cap f^{-1}(A)\right\} \\&\ge {\mathbb {P}}\left\{ f_{\omega }(x) \in \Delta ,f_{\omega }(x) +\omega _{1} \in \Delta \cap f^{-1}(A)\right\} \\  &= \int _{[-\sigma ,\sigma ]}1_{\Delta }\left( f(x+\omega _0)\right) {\mathbb {P}}\left\{ f(x+\omega _0)+\omega _1 \in f^{-1}(A) \cap \Delta \right\} d\omega _0 \\  &\ge \int _{[-\sigma ,\sigma ]}1_{\Delta }\left( f(x+\omega _0)\right) \inf _{y \in \Delta } {\mathbb {P}}\left\{ y+\omega _1 \in f^{-1}(A) \cap \Delta \right\} d\omega _0 \\  &\ge {\mathbb {P}}\left\{ f_{\omega }(x) \in \Delta \right\} \inf _{y \in \Delta }{\mathbb {P}}\left\{ y+\omega _0 \in f^{-1}(A) \cap \Delta \right\} . \end{aligned}$$

In the last line, the first term is greater than zero due to (6.5), the second term is positive due to the fact that $\sigma > \frac{1}{R}= |\Delta |$. This concludes the proof. $\square$

As a consequence of Lemma 6.3, the RDS $f^n_{\omega }$ has an unique ergodic measure $\mu$. The absolute continuity of $\mu$ follows from the fact that the transition probabilities $A \in \mathcal {B}({\mathbb {S}}^1) \mapsto {\mathbb {P}}(f_{\omega }(x) \in A)$ are absolutely continuous with respect to Lebesgue for all $x \in {\mathbb {S}}^1$ [17, chapter 1].

It remains to prove that $\log |df_{\omega }(x)|$ is integrable with respect to ${\mathbb {P}}\otimes \mu$. Observe that *df*(*x*) is uniformly bounded from above and is bounded away from 0 everywhere but in a small neighbourhood of the critical set $\mathcal {C}$, in which *df*(*x*) approaches 0. As a consequence, we only need to check that the integral of $\log |df_{\omega }(x)|$ with respect to ${\mathbb {P}}\otimes \mu$ is bounded from below. Due to the regularity of $\mathcal{S}\mathcal{C}$, there exists a constant $B \ge 1$ such that, for all *x* in a small neighbourhood $\mathcal {N}(\mathcal {C})$ of $\mathcal {C}$

$$\begin{aligned} |df(x)| \ge B \textrm{dist}(x,\mathcal {C}). \end{aligned}$$

Using this fact, we see that, there exists a constant $D_1 >0$ such that for all $x \in {\mathbb {S}}^1$

$$\begin{aligned} \int _{\Omega }\log |df(x+\omega _0)|d{\mathbb {P}}(\omega _0)&\ge \int _{B(x,\sigma )} \log \left| df(z)\right| dz\\  &\ge \int _{B(x,\sigma ){\setminus }\mathcal {N}(\mathcal {C})}\inf _{ x \in {\mathbb {S}}^1\setminus \mathcal {N}({\mathcal {C})}}\log |df(x)| \\&\quad + \int _{\mathcal {N}(\mathcal {C})} \log B \textrm{dist}(z,\mathcal {C})dz \\&\ge D_1 + \int _{\mathcal {N}(\mathcal {C})} \log \textrm{dist}(z,\mathcal {C})dz. \end{aligned}$$

Therefore,

$$\begin{aligned} \int _{{\mathbb {S}}^1\times \Omega }\log |df(x+\omega _0)|d\mu (x)d{\mathbb {P}}(\omega _0)&= \int _{{\mathbb {S}}^1}\left[ \int _{\Omega } \log \left| df(x+\omega _0)\right| d{\mathbb {P}}(\omega )\right] dx \\&\ge D_1+ \int _{\mathcal {N}(\mathcal {C})} \log \textrm{dist}(x,\mathcal {C})dx>-\infty , \end{aligned}$$

which concludes the proof. $\square$

### Full Branch Expanding Property

We have proved that Hypotheses (H1) and (H2) are fulfilled. Note also that Hypothesis (H6) is trivially satisfied. Indeed, let $G_{\omega }:= G-\omega _0$ be the super expanding region of $f_{\omega }$ and consider also the sets $\Delta _{\omega }:= \Delta -\omega _0$, for $\omega \in \Omega$. Then, $\Delta _{\omega } \subset G_{\omega }$ and $f_{\omega }(\Delta _{\omega })= {\mathbb {S}}^1$, for all $\omega \in \Omega$, due to Proposition 6.4. Now we prove that (H5) is also fulfilled.

Recall that (see Definition 2.4) an interval *I* is $(\omega ,\kappa _1,K)$-full branch expanding if there exist an interval $J \subset I$ and an integer $i \le K$ such that $f^i_{\omega }(J)= {\mathbb {S}}^1$ and $\left| df^i_{\omega }\vert _{J} \right| >\kappa _1$.

The main result of this subsection is the following

#### Proposition 6.4

Let $f^n_{\omega }$ be a $(\sigma ,R)$-predominantly expanding RDS. Then, Hypothesis (H5) is fulfilled, namely for sufficiently small $\eta >0$ there exists numbers $K \in {\mathbb {N}}$, and $\kappa _1 \ge 1$ such that there is a positive probability, bounded away from zero, that any interval whose diameter lies in $(\frac{\eta }{4},4\eta )$ is $(\omega ,\kappa _1,K)$-full branch expanding. In particular, (2.7) follows.

The proof is divided into three steps.

- In Lemma 6.5, we use (H6) to prove that for all $k \in {\mathbb {N}}$ and all $\omega \in \Omega$ there exists a random interval $J=J(\omega _0,\dots ,\omega _{k-1})$ such that *J* is $(\omega ,k,R^k)$-full branch expanding and $|J| \le R^{-k}$.
- In Lemma 6.7, we deduce from Lemma 6.5 uniform bounds on the probability that, given any $\eta _1>0$, there exists $K \in {\mathbb {N}}$ such that any interval *I* contained in the set $\Delta$ introduced in (6.1), with $|I| \ge \eta _1$, is $(\omega ,K,R^{K})$-full branch expanding;
- We combine Lemma 6.5 and Lemma 6.7, together with Proposition 6.1, to conclude the proof.

#### Lemma 6.5

For every $k \in {\mathbb {N}}$, there exist $\gamma _k \in (0,1)$ and a set of noise realizations $H_k$ of the form

6.6 $$\begin{aligned} H_k:=[-\sigma ,\sigma ]\times H'(\omega _1,\dots ,\omega _{k-1}) \end{aligned}$$

satisfying ${\mathbb {P}}(H_k)>\gamma _k$ and the property that for every $\omega \in H_k$ there exists a random interval $J_k=J_k(\omega _0,\dots ,\omega _{k-1})$ such that $|J_k| \le R^{-k}$, $f^i_{\omega }(J_k) \subset \Delta _{\theta ^{i}(\omega )}$ for all $i= 1,\dots ,k-1$ and $f^k_{\omega }(J_k)={\mathbb {S}}^1$, where $\Delta _{\omega }:= \Delta -\omega _0$, and $\Delta$ is as in (6.1). In particular, $J_k$ is $(\omega ,k,R^k)$-full branch expanding.

#### Proof

We first state, without proof, the following useful fact. $\square$

#### Lemma 6.6

Let $Q \subset {\mathbb {S}}^1$ be an interval such that $|Q|\le \frac{1}{R}$. Then

6.7 $$\begin{aligned} {\mathbb {P}}\left\{ f^{-1}(Q+\omega _0)\cap \Delta \,\,\text {is connected} \right\} \ge \frac{1}{4R\sigma }. \end{aligned}$$

We can then proceed with the proof of Lemma 6.5. Let $\bar{f}$ be the lifting of *f* and $\bar{\Delta }$ be a lifting of $\Delta$ such that $\bar{\Delta } \subset [0,1)$. Let us write $\bar{\Delta }:=[a,b]$, with $0\le a < b \le 1$ and $b-a\le \frac{1}{R}$. Without loss of generalities, let us assume that $\bar{f}(a)=0$ and $\bar{f}(b)=1$. Fix $k \in {\mathbb {N}}$ and define $J_1:= \Delta _{\theta ^{k-1}(\omega )}$, so that $f(J_1+\omega _{k-1})= {\mathbb {S}}^1$. Note that $J_1$ is connected by definition. Now take $J_2 \subset \Delta _{\theta ^{k-2}(\omega )}$ such that $f( J_2+\omega _{k-2})=J_1$. It follows that

$$\begin{aligned} J_2:= f^{-1}(J_1)\cap \Delta -\omega _{k-2}. \end{aligned}$$

Note that since $J_1 = \Delta -\omega _{k-1}$, we can average on $\omega _{k-1}$ and conclude, by Lemma 6.6, that $J_2$ is connected with probability greater than $\frac{1}{4R\sigma }$.. Proceeding inductively, we construct a sequence of random intervals $\{J_i\}_{i=1,\dots ,k}$ such that $J_i \subset \Delta _{\theta ^{k-i}(\omega )}$, $f(J_i+\omega _{k-i})=J_{i-1}$ for $2 \le i \le k-1$, $f(J_1+\omega _{k-1})= {\mathbb {S}}^1$ and all intervals $\{J_i\}_{i =1}^n$ are connected as long as the noise realizations $\{\omega _i\}_{i=1}^{k-1}$ are chosen so that

$$\begin{aligned} J_i:= f^{-1}(J_{i-1}) \cap \Delta -\omega _{k-i} \end{aligned}$$

is connected for all $i=2,\dots , k-1$. By equation (6.7), this happens with probability larger than $\left( \frac{1}{4R\sigma }\right) ^{k-1}$. The other properties required for the random sets $J_{i}$ follow by construction. $\square$

In the following Lemma, we show that intervals in $\Delta$ are full branch expanding.

#### Lemma 6.7

For any $\eta _1>0$, there exists $K_1 \in {\mathbb {N}}$ and $\iota _1 \in (0,1)$ such that, if $|I| \ge \eta _1$ and $I \subset \Delta$, then

$$\begin{aligned} {\mathbb {P}}\left\{ I \,\,\text {is} \,\, (\omega ,K_1,R^{K_1})\text {-full branch expanding}\right\} \ge \iota _1. \end{aligned}$$

#### Proof

Let $K_1$ be such that $\eta _1 \gg R^{-K_1}$. Let $H_k \subset \Omega$ be the set of noise realization defined in (6.6). Now consider the set

$$\begin{aligned} \tilde{H}_k:= \theta ^{-1}(H_k), \end{aligned}$$

so that for all $\omega \in \tilde{H}_k$ there exists an interval $J=J(\omega _1,\dots ,\omega _{K_1})$ such that $f^{K_1-1}_{\theta (\omega )}(J)= {\mathbb {S}}^1$, $f^i_{\theta (\omega )}(J) \subset \Delta _{\theta ^{i+1}(\omega )}$ and $|J| \le R^{-K_1+1}$.

Let $\tilde{J}= \tilde{J}(\omega _1,\dots ,\omega _{K_1}) \subset \Delta$ be such that $f(\tilde{J})= J$. Since $\tilde{J} \subset \{|df|> R \}$, we have that $\left| \tilde{J}\right| \le R^{-K_1}<<\eta _1$. Let $\tilde{H}_{k+1}$ denote the set of noise realization such that $\tilde{J}$ is connected. Observe that

$$\begin{aligned} \left\{ I \,\,\text {is} \,\, (\omega ,K_1,R^{K_1})\text {-full branch expanding}\right\} \supset \left\{ I+\omega _0 \supset \tilde{J}\,\,\text {and}\,\, \tilde{J}\,\text {is connected} \right\} . \end{aligned}$$

As a consequence, we see that

6.8 $$\begin{aligned}  &   {\mathbb {P}}\left\{ I \,\,\text {is} \,\, (\omega ,K_1,R^{K_1})\text {-full branch expanding}\right\} \ge {\mathbb {P}}\left\{ I+ \omega _0 \supset \tilde{J}\,\,\text {and} \,\, \tilde{J} \,\,\text {is connected}\right\} \nonumber \\    &   \displaystyle \quad \ge \int _{\tilde{H}_{k+1}} \left[ \int {\mathbb {P}}\left\{ I+\omega _0\ \supset \tilde{J} \right\} d{\mathbb {P}}(\omega _0)\right] d{\mathbb {P}}(\omega _1,\dots ,\omega _{K_1}). \end{aligned}$$

Since both *I* and $\tilde{J}$ are contained in $\Delta$ and $\sigma> \frac{1}{R}> |\Delta |$ due to the $(\sigma ,R)$-predominant expansion, there exists $\iota _1>0$ such that

$$\begin{aligned} {\mathbb {P}}\left\{ I+\omega _0\,\,\text {contains}\,\, \tilde{J} \right\} >\iota _1, \end{aligned}$$

for all $\omega \in \tilde{H}_{k+1}$. By (6.8) and (6.6) this concludes the proof. $\square$

#### Proof of Proposition 6.4

Let $C:= \sup ||f||_{\mathcal {C}^1\setminus \mathcal{S}\mathcal{C}}$. Define $\eta :=\frac{\gamma }{2C}$. Let *I* be an interval such that $|I| \in (\frac{\eta }{4},4\eta )$. In particular, $I \subset B(x_I,2\eta )$, where $x_I$ is the middle point of *I*. Using (6.4) with $x=x_I$, we see that

$$\begin{aligned} {\mathbb {P}}\left\{ x+\omega _0 \in E\,\,\text {and} \,\, f_{\omega }\left( I\right) \subset \Delta \right\} >q. \end{aligned}$$

Note that, due to the expansion in *E* (for the definition refer to equation (6.3)), the event $\left\{ x+\omega _0 \in E\,\,\text {and} \,\, f_{\omega }\left( I\right) \subset \Delta \right\}$ is contained in the event

6.9 $$\begin{aligned} E(I):= \left\{ f_{\omega }(I) \subset \Delta ,\, |f_{\omega }(I)|\ge \frac{\eta }{4}\,\text {and} \, \left| df_{\omega }\vert _{I} \right| >1 \right\} \end{aligned}$$

Hence,

6.10 $$\begin{aligned} {\mathbb {P}}\left( E(I) \right) >q. \end{aligned}$$

By Lemma 6.7 with $\eta _1=\frac{\eta }{4}$, we find $K_1$ and $\iota _1$ such that

6.11 $$\begin{aligned} \inf _{\{I \subset \Delta ,|I| \ge \frac{\eta }{4}\}} {\mathbb {P}}\left\{ I \,\,\text {is} \,\, (\omega ,K_1,R^{K_1})\text {-full branch expanding}\right\} > \iota _1. \end{aligned}$$

As a consequence, due to (6.9)

$$\begin{aligned}&{\mathbb {P}}\left\{ I\,\text {is}\, (\omega ,K_1+1,R^{K_1})\,\text {full branch expanding} \ \right\} \\&\quad \ge \int _{E(I)} {\mathbb {P}}\left\{ f(I+\omega _0)\,\text {is}\,(\theta (\omega ),K_1,R^{K_1}) \text {full branch expanding}\right\} \\&\quad d{\mathbb {P}}(\omega _0) \ge q\iota _1, \end{aligned}$$

which concludes the proof. $\square$

## Large Deviation Estimates for Slow Recurrence to the Critical Set and for the Lyapunov Exponent

### Slow Recurrence to the Critical Set

In this subsection, we show that $(\sigma ,R)$-predominantly expanding RDSs satisfy Hypothesis (H4). Let $f^n_{\omega }$ be a $(\sigma ,R)$-predominantly expanding random dynamical system with *f* as a generating map. The singular set $\mathcal{S}\mathcal{C}$ of *f* has the form

$$\begin{aligned} \mathcal{S}\mathcal{C}:= \left\{ a_0,a_1,\dots ,a_{m-1} \right\} , \end{aligned}$$

where $m=|\mathcal{S}\mathcal{C}|$ denotes the cardinality of the singular set. Using the same notation as in Hypothesis (H4), let

$$\begin{aligned} Z_n(\delta ,\omega ,x)&:= \sum _{i=0}^{n-1}- \log \left( \textrm{dist}_{\delta }\left( f^i_{\omega }(x),\mathcal{S}\mathcal{C}_{\theta ^i(\omega )}\right) \right) . \end{aligned}$$

By Birkhoff ergodic theorem, we see that

$$\begin{aligned} \lim _{n \rightarrow \infty } \frac{Z_n(\omega ,x,\delta )}{n} = \int _{{\mathbb {S}}^1\times \Omega } -\log \left( \textrm{dist}_{\delta } \left( x,\mathcal{S}\mathcal{C}_{\omega }\right) \right) d\mu (x)d{\mathbb {P}}(\omega ) \qquad {\mathbb {P}}\otimes \mu \,\, \text {a.s.} \end{aligned}$$

The main result of this subsection is the following

#### Theorem 7.1

Let $f^n_{\omega }$ be a $(\sigma ,R)$-predominantly expanding RDS and let

$$\begin{aligned} H(\delta ):=\sqrt{\delta }\left( 1+\log \left( \frac{1}{\delta }\right) \right) . \end{aligned}$$

Then, there exists $p \in (0,1)$ such that

$$\begin{aligned} {\mathbb {P}}_x( Z_n(\omega ,x,\delta )> n H(\delta ) ) \le p^n \qquad \forall n \ge 0, \end{aligned}$$

for $\delta$ small enough. In particular, Hypothesis (H4) is fulfilled.

#### Proof

By the Markov inequality, for $1<\zeta < e$, we have

$$\begin{aligned} {\mathbb {P}}\left( Z_n(\omega ,x,\delta )> n H(\delta ) \right) \le \frac{{\mathbb {E}} \left[ \prod _i \zeta ^{-\log \left( \textrm{dist}_{\delta }\left( f^i_{\omega }(x), \mathcal{S}\mathcal{C}_{\theta ^i(\omega )}\right) \right) }\right] }{\zeta ^{H(\delta )n}}. \end{aligned}$$

In order to simplify the notation, we write $b_i(\omega ,x,\delta ):=-\log \big (\textrm{dist}_{\delta }\big (f^i_{\omega }(x), \mathcal{S}\mathcal{C}_{\theta ^i(\omega )}\big )\big )$. Let $\mathcal {F}_{n}$ denote the Sigma algebra generated by the projection of $\omega \in \Omega$ to the first *n* coordinates, i.e.

$$\begin{aligned} \mathcal {F}_n:= \sigma \left( \omega _0,\dots ,\omega _{n-1}\right) . \end{aligned}$$

Note that

7.1 $$\begin{aligned} {\mathbb {E}}\left[ \prod _{i\le n} \zeta ^{b_i(\omega ,x,\delta )} \right] = {\mathbb {E}}\left[ \prod _{i\le n-1}\zeta ^{b_i(\omega ,x,\delta )}{\mathbb {E}}\left[ \zeta ^{b_n(\omega ,x,\delta )}|\mathcal {F}_{n-1}\right] (\omega )\right] \end{aligned}$$

Due to the the identity $\mathcal{S}\mathcal{C}_{\omega }=\mathcal{S}\mathcal{C}-\omega _0$ and the fact that $\mathcal{S}\mathcal{C}_{\theta ^n(\omega )}$ depends explicitly on $\omega _n$, whilst $f^n_{\omega }(x)$ depends on $\omega _0,\dots ,\omega _{n-1}$, we have,

$$\begin{aligned} {\mathbb {E}}\left[ \zeta ^{b_n(\omega ,x,\delta )}|\mathcal {F}_{n-1}\right] (\omega )&= \int _{(-\sigma ,\sigma )} \zeta ^{-\log \left( \textrm{dist}_{\delta }(f^n_{\omega }(x)+\omega _n,\mathcal{S}\mathcal{C})\right) } d{\mathbb {P}}(\omega _n)\\  &= \frac{1}{2\sigma } \int _{{\mathbb {S}}^1} \zeta ^{-\log \left( \textrm{dist}_{\delta }(y,\mathcal{S}\mathcal{C})\right) } 1_{B (f^{n}_{\omega }(x),\sigma )}(y)dy \\  &= \frac{1}{2\sigma } \int _{{\mathbb {S}}^1} 1_{B(f^n_{\omega }(x),\sigma )}(y) \left( \frac{1}{\textrm{dist}_{\delta }(y,\mathcal{S}\mathcal{C})}\right) ^{\log (\zeta )} \mathrm dy \\  &= \frac{1}{2\sigma }\int _{{\mathbb {S}}^1{\setminus } B(\mathcal{S}\mathcal{C},\delta )}1_{B (f^n_{\omega }(x),\sigma )}dy\\  &\quad + \frac{1}{2\sigma }\int _{B(\mathcal{S}\mathcal{C},\delta )}1_{B(f^{n}_{\omega }(x),\sigma )}(y) \left( \frac{1}{\textrm{dist}_{\delta }(y,\mathcal{S}\mathcal{C})}\right) ^{\log (\zeta )}d(y) \\&\le 1 + \frac{2m}{\sigma } \int _{(0,\delta )}\left( \frac{1}{x}\right) ^{\log (\zeta )} dx \\&= 1 + \frac{2m}{\sigma }\frac{\delta ^{1-\log (\zeta )}}{1-\log (\zeta )}. \end{aligned}$$

Substituting this estimate into (7.1), we achieve by induction in *n* that

7.2 $$\begin{aligned} {\mathbb {P}}\left( Z_n(\omega ,x,\delta )> n H(\delta ) \right) \le \left( \frac{1 + \frac{2m}{\sigma }\frac{\delta ^{1-\log (\zeta )}}{1-\log (\zeta )}}{\zeta ^{H(\delta )}}\right) ^n. \end{aligned}$$

Furthermore,

$$\begin{aligned} \frac{d \zeta ^{H(\delta )}}{d\delta }&= \log (\zeta ) \zeta ^{H(\delta )} \left( \frac{1}{2\sqrt{\delta }} + \frac{\log (\frac{1}{\delta })}{2\sqrt{\delta }} - \frac{1}{\sqrt{\delta }} \right) \\  &= \frac{1}{2\sqrt{\delta }}\log (\zeta ) \zeta ^{H(\delta )} \left( \log \left( \frac{1}{\delta }\right) -1\right) \\  &> \frac{1}{2\sqrt{\delta }}\log (\zeta ) \,\, \text {as} \, \delta \rightarrow 0. \end{aligned}$$

Thus, for sufficiently small $\delta$,

7.3 $$\begin{aligned} \zeta ^{H(\delta )}-1 > \lim _{t \rightarrow 0} \int _t^{\delta } \log (\zeta ) \frac{1}{2\sqrt{x}}dx = \log (\zeta ) \sqrt{\delta }. \end{aligned}$$

Choose $\zeta$ sufficiently close to one so that $\frac{1}{2}<1-\log (\zeta )$. Then, if $\delta$ is small enough, we have that

7.4 $$\begin{aligned} \log (\zeta )\sqrt{\delta } > \frac{2m}{\sigma }\frac{\delta ^{1-\log (\zeta )}}{1-\log (\zeta )}. \end{aligned}$$

As a consequence,

$$\begin{aligned} p := \left( \frac{1 + \frac{2m}{\sigma }\frac{\delta ^{1-\log (\zeta )}}{1-\log (\zeta )}}{\zeta ^{H(\delta )}}\right) <1, \end{aligned}$$

which, by (7.2), concludes the proof. $\square$

### Positivity of Lyapunov Exponent and Large Deviations

The main result of this subsection is the following

#### Proposition 7.2

Let $f^n_{\omega }$ be $(\sigma ,R)$-predominantly expanding RDS. Then, the Lyapunov exponent $\lambda$ is positive and satisfies

7.5 $$\begin{aligned} \lambda > Z(h), \end{aligned}$$

where *Z*(*h*) is given by (2.15) in Definition 2.8. Furthermore, if $\alpha >0$ is taken small enough so that

7.6 $$\begin{aligned} \bar{Z}(h):= \log (R)(1-h)+\frac{\alpha +1}{2\sigma }\int _{C} \log |df(z)|dz>0, \end{aligned}$$

where *C* is the contracting region of *f*, then there exists a constant $D \ge 1$ and $q \in (0,1)$ such that

7.7 $$\begin{aligned} \sup _{x \in {\mathbb {S}}^1} {\mathbb {P}}\left( \sum _{i=0}^{n-1}\log \left| df_{\theta ^i(\omega )}(f^i_{\omega }x)\right| <\bar{Z}(h)n\right) \le Dq^n. \end{aligned}$$

In particular, Hypothesis (H3) is fulfilled.

#### Proof

By Proposition 6.2, the Markov chain associated with the RDS $f^n_{\omega }$ has an unique ergodic stationary measure $\mu$ such that $\log \left| df(x+\omega _0)\right| \in L^1({\mathbb {P}}\otimes \mu )$. As a consequence,

7.8 $$\begin{aligned} \lambda = \int _{{\mathbb {S}}^1}\int _{\Omega }\log \left| df(x+\omega _0)\right| d\mu (x)d{\mathbb {P}}(\omega ) \ge \inf _{x \in {\mathbb {S}}^1}\int _{\Omega }\log \left| df(x+\omega _0)\right| d{\mathbb {P}}(\omega )\quad \nonumber \\ \end{aligned}$$

Furthermore, equations (2.14) and (2.15) imply that for any $x \in {\mathbb {S}}^1$ we have

$$\begin{aligned} \int _{\Omega }\log |df(x+\omega _0)|d{\mathbb {P}}(\omega )&= \frac{1}{2\sigma }\int _{B(x,\sigma ) \cap G}\log |df(z)| dz + \frac{1}{2\sigma }\int _{B(x,\sigma )\setminus G} \log |df(z)|dz \\&\ge \log (R)(1-h) + \frac{1}{2\sigma }\int _{B(x,\sigma )\setminus G}\log |df(z)| dz>Z(h)>0. \end{aligned}$$

It remains to establish the large deviations estimate (7.7). First, in order to simplify the notation, let $V:=-\int _C\log (|df(x)|)dx>0$. Then

7.9 $$\begin{aligned} \bar{Z}(h)= (1-h)\log (R)-\frac{\alpha +1}{2\sigma }V. \end{aligned}$$

For sufficiently large $l \in {\mathbb {N}}$, we consider the sets

7.10 $$\begin{aligned} P(k):= \left\{ x \in {\mathbb {S}}^1 \mid R^{-\frac{k}{l}}>|df(x)|>R^{-\frac{(k+1)}{l}} \right\} . \end{aligned}$$

Then $\{ P(k)\}_{k \ge -l}$ is a partition of ${\mathbb {S}}^1 {\setminus } G$ whose diameter is small if *l* is large and the sets $\{P(k)\}_{k \ge 0}$ are a partition of *C* with the same properties. Then, for any sequence $\{x_k\}_{k \ge 0}$ such that $x_k \in P(k)$ for *l* large enough we have

$$\begin{aligned} \sum _{k \ge 0} \log |df(x_k)|\textrm{Leb}(P(k)) \approx -V. \end{aligned}$$

Furthermore, as a consequence of the definition of *P*(*k*) in (7.10), we have that

$$\begin{aligned} \left| \sum _{k \ge 0} \log |df(x_k)|\textrm{Leb}(P(k))- \sum _{k \ge 0} \log (R^{-\frac{(k+1)}{l}})\textrm{Leb}(P(k)) \right| \le \log \left( R^{-\frac{1}{l}}\right) \textrm{Leb}(C). \end{aligned}$$

We can choose *l* large enough such that

7.11 $$\begin{aligned} \frac{\sum _{k\ge 0} \frac{(k+1)}{l}\textrm{Leb}(P(k))}{2\sigma }< \left( \frac{1}{2\sigma \log (R)}+ \frac{\alpha }{4\sigma \log (R)}\right) V. \end{aligned}$$

Fix $n \in {\mathbb {N}}$ and $x \in {\mathbb {S}}^1$. Given $k \ge -l$ let $\tilde{P}_{n,k}(\omega ,x)$ be the number of points among $x+\omega _0, \dots , f^{n-1}_{\omega }(x)+\omega _{n-1}$ that belong to *P*(*k*):

7.12 $$\begin{aligned} \tilde{P}_{n,k}(\omega ,x):= \text {card}\{i \in \{0,\dots , n-1 \} \mid f^i_{\omega }(x) +\omega _i \in P(k) \}, \end{aligned}$$

Analogously, we define

$$\begin{aligned} \tilde{G}_n(\omega ,x)&:= \text {card}\{i \in \{0,\dots , n-1 \} \mid f^i_{\omega }(x)+\omega _i \in G \}. \end{aligned}$$

Recall that *G* is the super-expanding region of *f* and $\bigcup _{k \ge -l}P(k) = {\mathbb {S}}^1 {\setminus } G$.

By definition of *G* and *P*(*k*), we have

7.13 $$\begin{aligned} S_n(\omega ,x):  &   =\sum _{i=0}^{n-1}\log |df_{\theta ^i(\omega )}(f^i_{\omega }(x))| \ge \tilde{G}_{n}(\omega ,x)\log (R) \nonumber \\  &   \quad - \sum _{k =0}^{\infty }\tilde{P}_{n,k}(\omega ,x) \frac{(k+1)}{l}\log (R). \end{aligned}$$

Given $x \in {\mathbb {S}}^1$, consider the set of noise realizations

$$\begin{aligned} A_n(x) \!:=\! \left\{ \tilde{G}_n(\omega ,x) \!-\! \sum _{k \ge 0} \tilde{P}_{n,k}(\omega ,x) \frac{(k+1)}{l} \!<\! \left( 1-h-\left( \frac{1+\alpha }{2\sigma \log (R)}\right) V\right) n \right\} . \end{aligned}$$

Note that by (7.9) and (7.13) we have

7.14 $$\begin{aligned} \left\{ S_n(\omega ,x)< \bar{Z}(h)n \right\} \subset A_n(x). \end{aligned}$$

In order to estimate the measure of $A_n(x)$, we introduce the set $A^1_n(x)$ of all noise realizations for which the orbit of *x* enters one of the sets *P*(*k*) for $k \ge n$ at least once:

$$\begin{aligned} A^1_n(x)&:= \left\{ \exists k \ge n \mid \tilde{P}_{n,k}(\omega ,x) >0 \right\} . \end{aligned}$$

We further consider the set of noise realizations for which the orbit of *x* visits *G* with frequency smaller than $1-h- \frac{\alpha }{8\sigma \log (R)}V$:

$$\begin{aligned} A^2_n(x) := \left\{ \tilde{G}_{n}(\omega ,x)\le \left( 1-h-\frac{\alpha }{8\sigma \log (R)}V\right) n \right\} . \end{aligned}$$

The set $A_n(x)$ can be decomposed as:

7.15 $$\begin{aligned} A_n(x):= A^3_n(x) \sqcup \left( A_n(x) \cap \left( A^1_n(x) \cup A^2_n(x)\right) \right) , \end{aligned}$$

where

7.16 $$\begin{aligned} \begin{array}{ll} A^3_n(x) & = A_n(x){\setminus }\left( A^1_n(x)\cup A^2_n(x)\right) \\ & = \left\{ \sum \limits _{k = 0}^{n-1} \tilde{P}_{n,k}(\omega ,x) \left( \frac{k+1}{l}\right) > \tilde{Z}n \right\} , \end{array} \end{aligned}$$

and

7.17 $$\begin{aligned} \tilde{Z}:= \left( \frac{3\alpha }{8\sigma \log (R)}+\frac{1}{2\sigma \log (R)}\right) V. \end{aligned}$$

As

$$\begin{aligned} {\mathbb {P}}(A_n(x)) \le {\mathbb {P}}\left( A^1_n(x)\right) + {\mathbb {P}}(A^2_n(x)) + {\mathbb {P}}(A^3_n(x)), \end{aligned}$$

Proposition 7.2 is to follow from the estimates on the measure of $A^1_n(x)$, $A^2_n(x)$ and $A^3_n(x)$, in Lemmas 7.3, 7.4 and 7.5 below. $\square$

#### Lemma 7.3

There exists a $D_0 \ge 1$, such that, for all $n \ge 0$,

$$\begin{aligned} {\mathbb {P}}\left( A^1_n(x)\right) \le D_0hR^{-\frac{n}{2l}}. \end{aligned}$$

#### Proof

Let

$$\begin{aligned} \tilde{P}(n):= \bigcup _{k \ge n}P(k), \end{aligned}$$

then

$$\begin{aligned} {\mathbb {P}}\left( A^1_n(x)\right) \le \sum _{i=0}^{n-1} {\mathbb {P}}\left( f^i_{\omega }(x)+x_i \in \tilde{P}(n) \right) \le \sum _{i=0}^{n-1} \sup _{y \in {\mathbb {S}}^1}{\mathbb {P}}\left( y+\omega _i \in \tilde{P}(n)\right) . \end{aligned}$$

By item (1) Definition 2.8, the critical set of *f* is non-degenerated. Then, there exists a constant $C \ge 1$ such that $\textrm{Leb}(P(k))\le C R^{-\frac{n}{l}}$, for all $n \ge 0$. As a consequence, we have that

$$\begin{aligned} {\mathbb {P}}\left( y+\omega _i \in \tilde{P}(n) \right) \le \frac{\textrm{Leb}(\tilde{P}(n))}{2\sigma }&\le D R^{-\frac{n}{l}}, \end{aligned}$$

which implies that

$$\begin{aligned} {\mathbb {P}}\left( A^1_n(x)\right) \le DnR^{-\frac{n}{l}} \le D_0R^{-\frac{n}{2l}}, \end{aligned}$$

for some $$D_0 \ge 1$$. $$\square $$

#### Lemma 7.4

There exists $\tilde{q}_1 \in (0,1)$ such that, for all $n \ge 0$

$$\begin{aligned} {\mathbb {P}}\left( A^2_n(x)\right) \le \tilde{q}_1^n. \end{aligned}$$

#### Proof

Observe that $A^2_n(x)$ can be rewritten as

$$\begin{aligned} A^2_{n}(x)= \left\{ \sum _{i=0}^{n-1}1_{{\mathbb {S}}^1{\setminus } G}(f^i_{\omega }(x)+\omega _i)>\left( h+ \frac{\alpha }{4}V\right) n \right\} . \end{aligned}$$

By the Markov inequality, for any $\zeta >1$, we obtain that

$$\begin{aligned} {\mathbb {P}}\left( A^2_{n}(x)\right) \le \frac{{\mathbb {E}}\left[ \zeta ^{\sum _{i=0}^{n-1}1_{{\mathbb {S}}^1\setminus G}\left( f^i_{\omega }(x)+\omega _i\right) }\right] }{\zeta ^{\left( h+ \frac{\alpha }{4}V\right) n}}. \end{aligned}$$

We may rewrite the upper term as

$$\begin{aligned} {\mathbb {E}}\left[ \zeta ^{\sum _{i=0}^{n-1}1_{{\mathbb {S}}^1\setminus G}(f^i_{\omega }(x)+\omega _i)}\right]&= {\mathbb {E}}\left[ \prod _{i=0}^{n-1} {\zeta ^{1_{{\mathbb {S}}^1\setminus G}(f^i_{\omega }(x)+\omega _i)}}\right] \\&= \int _{[-\sigma ,\sigma ]^n} \prod _{i=0}^{n-1} \zeta ^{1_{{\mathbb {S}}^1\setminus G}(f^i_{\omega }(x)+\omega _i)} d{\mathbb {P}}(\omega _0,\dots \omega _{n-1}) \\&= \int _{[-\sigma ,\sigma ]^{n-1}}\prod _{i=0}^{n-2} \zeta ^{1_{{\mathbb {S}}^1\setminus G}(f^i_{\omega }(x)+\omega _i)}\\&\quad \left[ \int _{\omega _{n-1}} \zeta ^{1_{{\mathbb {S}}^1\setminus G}(f^{n-1}_{\omega }(x)+\omega _{n-1})}d{\mathbb {P}}(\omega _{n-1})\right] \\  &\qquad d{\mathbb {P}}(\omega _0,\dots ,\omega _{n-2}). \end{aligned}$$

Now observe that, given $\omega _0,\dots ,\omega _{n-2}$ fixed, we have that

$$\begin{aligned}&\int _{[-\sigma ,\sigma ]} \zeta ^{1_{{\mathbb {S}}^1\setminus G}(f^{n-1}_{\omega }(x)+\omega _{n-1})}d{\mathbb {P}}(\omega _{n-1}) = \zeta \frac{\textrm{Leb}(\omega _n \mid f^{n-1}_{\omega }(x)+\omega _{n-1} \notin G)}{2\sigma }\\&\quad + 1- \frac{\textrm{Leb}(\omega _n \mid f^{n-1}_{\omega }(x)+\omega _{n-1} \notin G)}{2\sigma }. \end{aligned}$$

Furthermore, by (2.14) we have

$$\begin{aligned} \int _{\omega _{n-1}} \zeta ^{1_{{\mathbb {S}}^1\setminus G}(f^{n-1}_{\omega }(x)+\omega _{n-1})}d{\mathbb {P}}(\omega _{n-1}) \le \zeta h + 1-h. \end{aligned}$$

Repeating this argument recursively we obtain

$$\begin{aligned} {\mathbb {E}}\left[ \zeta ^{\sum _{i=0}^{n-1}1_{{\mathbb {S}}^1\setminus G}(f^i_{\omega }(x)+\omega _i)}\right] \le (\zeta h + 1-h)^n. \end{aligned}$$

Hence, for any $\zeta >1$

$$\begin{aligned} {\mathbb {P}}\left( A^2_n(x)\right) \le \left( \frac{h\zeta + 1-h}{\zeta ^{h+\frac{\alpha }{4}V}}\right) ^n. \end{aligned}$$

Taking $\tilde{q}_1=\left( \frac{\zeta h+ 1-h}{\zeta ^{h+\frac{\alpha }{4}V}}\right)$ for $\zeta$ close to 1 concludes the proof. $\square$

#### Lemma 7.5

There exists $\tilde{q}_2 \in (0,1)$ such that, for all $n \ge 0$

$$\begin{aligned} {\mathbb {P}}\left( A^3_n(x)\right) \le \tilde{q}_2^n. \end{aligned}$$

#### Proof

By the Markov inequality and (7.16), for all $\zeta >1$.

7.18 $$\begin{aligned} {\mathbb {P}}(A^3_n(x)) \le \frac{{\mathbb {E}}\left[ \zeta ^{ \sum _{k = 0}^{n-1} \tilde{P}_{n,k}(\omega ,x)\frac{k+1}{l}}\right] }{\zeta ^{n\tilde{Z}}}. \end{aligned}$$

Define for $$j = 0,\dots , n-1$$

$$\begin{aligned} M_j(\omega ,x) := \prod _{k=0}^{n-1}\zeta ^{\frac{k+1}{l}1_{P(k)}(f^j_{\omega }(x)+\omega _j)}, \end{aligned}$$

and let

$$\begin{aligned} \pi _j(A) = {\mathbb {P}}((\omega _0,\dots ,\omega _{j-1}) \in A) \qquad \forall A \subset \mathcal {B}([-\sigma ,\sigma ]^j). \end{aligned}$$

Then, by (7.12)

$$\begin{aligned} {\mathbb {E}}\left[ \zeta ^{ \sum _{k = 0}^{n-1} \tilde{P}_{n,k}(\omega ,x)\frac{(k+1)}{l}}\right]&= {\mathbb {E}}\left[ \prod _{k=0}^{n-1}\zeta ^{\tilde{P}_{n,k}(\omega ,x)\frac{(k+1)}{l}} \right] = {\mathbb {E}}\left[ \prod _{k=0}^{n-1}\prod _{j=0}^{n-1}\zeta ^{\frac{(k+1)}{l}1_{P(k)}(f^j_{\omega }(x)+\omega _j)} \right] \\&={\mathbb {E}}\left[ \prod _{j=0}^{n-1}\left[ \prod _{k=0}^{n-1} \zeta ^{\frac{(k+1)}{l}1_{P(k)}(f^j_{\omega }(x)+\omega _j)} \right] \right] = \int _{\Omega } \prod _{j=0}^{n-1} M_j(\omega ,x)d\pi _n. \end{aligned}$$

By Fubini’s theorem

7.19 $$\begin{aligned} \int _{\Omega } \prod _{j=0}^{n-1}M_j(\omega ,x)d\pi _{n}= \int _{[-\sigma ,\sigma ]^{n-1}} \prod _{j=0}^{n-2}{M_j(\omega ,x)}\left[ \int _{[-\sigma ,\sigma ]}M_{n-1}(\omega ,x) d{\mathbb {P}}(\omega _n)\right] d\pi _{n-1}.\nonumber \\ \end{aligned}$$

For given $(\omega _0,\dots ,\omega _{n-2}) \in [-\sigma ,\sigma ]^{n-1}$, we may rewrite

$$\begin{aligned}&\int _{[-\sigma ,\sigma ]}M_{n-1}(\omega ,x)d{\mathbb {P}}(\omega _{n-1}) = \int _{[-\sigma ,\sigma ]} \prod _{k=0}^{n-1} \zeta ^{\frac{k+1}{l}1_{P(k)}(f^{n-1}_{\omega }(x)+\omega _{n-1})}d{\mathbb {P}}(\omega _{n-1})\\&\le \sup _{y \in {\mathbb {S}}^1} \left\{ \int _{[-\sigma ,\sigma ]} \prod _{k=0}^{n-1} \zeta ^{\frac{k+1}{l}1_{P(k)}(y+\omega _{n-1})}{\mathbb {P}}(\omega _{n-1})\right\} \\  &\le \sup _{y \in {\mathbb {S}}^1} \left\{ 1- \frac{\textrm{Leb}\left( \cup _{k=0}^{n-1 }P(k) \cap B(y,\sigma )\right) }{2\sigma }+ \sum _{k=0}^{n-1} \zeta ^{\frac{(k+1)}{l}}\frac{\textrm{Leb}\left( P(k) \cap B(y,\sigma )\right) }{2\sigma } \right\} \\  &\le \sup _{y \in {\mathbb {S}}^1} \left\{ 1+ \sum _{k=0}^{n-1} (\zeta ^{\frac{(k+1)}{l}}-1)\frac{\textrm{Leb}\left( P(k) \cap B(y,\sigma )\right) }{2\sigma } \right\} \\  &\le 1+ \sum _{k=0}^{n-1} (\zeta ^{\frac{(k+1)}{l}}-1)\frac{\textrm{Leb}\left( P(k) \right) }{2\sigma } \end{aligned}$$

As a consequence,

7.20 $$\begin{aligned} \int _{\Omega } \prod _{j=0}^{n-1}M_j(\omega ,x)d\pi _{n-1}\le W(\zeta ) \int _{\Omega } \prod _{j=0}^{n-1}M_j(\omega ,x)d\pi _{n-2}, \end{aligned}$$

where

$$\begin{aligned} W(\zeta )= 1+ \sum _{k=0}^{n-1} (\zeta ^{\frac{(k+1)}{l}}-1)\frac{\textrm{Leb}\left( P(k)\right) }{2\sigma }. \end{aligned}$$

Repeating this argument, we obtain

7.21 $$\begin{aligned} {\mathbb {P}}(A^3_n(x)) \le \left( \frac{W(\zeta )}{\zeta ^{\tilde{Z}}}\right) ^n. \end{aligned}$$

Note that $W(1)=1$ and, by (7.11) and (7.17)

$$\begin{aligned} dW(1)&= \sum _{k=0}^{n-1} \frac{(k+1)}{l}\frac{\textrm{Leb}\left( P(k)\right) }{2\sigma }< \left( \frac{1}{2\sigma \log (R)}+\frac{\alpha }{4\sigma \log (R)}\right) V< \tilde{Z}. \end{aligned}$$

This implies that $W(\zeta )$ grows slower than $\zeta ^{\tilde{Z}}$ near $\zeta =1$. Therefore

$$\begin{aligned} q:= \left( \frac{W(\zeta )}{\zeta ^{\tilde{Z}}}\right) <1, \end{aligned}$$

for $\zeta >1$ sufficiently close to 1, which concludes the proof. $\square$

#### Remark 7.6

To facilitate future applications of the above result, it is important to highlight the proof of Proposition 7.5 requires only the use of Definition 2.8, items (1),  (2) and (3).

## Proof of Theorem 1.2

In this final section, we prove Theorem 1.2. It follows from Theorem 2.9 and the following proposition.

### Proposition 8.1

Let $f^n_{\omega }$ be the random dynamical system generated by random composition of the maps in (1.7), i.e. maps of the form

$$\begin{aligned} f_{\omega }(x):= L\sin (2\pi (x+\omega _0)) \, \,(\textrm{mod}\,1). \end{aligned}$$

Suppose $L \!\ge \! 3$. Then, $f^n_{\omega }$ is $(\sigma ,R)$-predominantly expanding for $R:= \max \{3,L^{\frac{1}{2}} \}$ and $\sigma > \frac{1}{R}+ c_1\frac{R}{\pi ^2\,L}$, with $c_1:= \frac{4\pi }{\sqrt{16 \pi ^2-1 }}$.

### Proof

Recall that $f^n_{\omega }$ is $(\sigma ,R)$-predominantly expanding if and only if the following two conditions are satisfied:

1. the circle map
  $$\begin{aligned} f(x):= L\sin (2\pi x)\,\, (\textrm{mod}\,1) \end{aligned}$$
  satisfies the conditions stated in Definition 2.8 (1);
2. the inequalities in (2.11) and (2.13) are satisfied.

Item (1) follows from the definition of *f*. To verify (2), we check the inequalities in (2.11) and (2.13). Equation (2.11) follows from the inequality

$$\begin{aligned} -\frac{c_1}{\pi ^2 L} \le \int _{C}\log |df(x)|dx, \end{aligned}$$

whilst (2.13) follows from (2.12) and the fact that

$$\begin{aligned} \textrm{Leb}\left( {\mathbb {S}}^1{\setminus }G\right)&= \textrm{Leb}\left\{ x \in {\mathbb {S}}^1 \mid |2\pi L \cos (2\pi x)|<R \right\} \\  &\le \textrm{Leb}\left\{ \bigcup _{\left\{ x_0= \frac{1}{4},\frac{3}{4}\right\} }\left\{ x \in {\mathbb {S}}^1 \mid \text {dist}(x,x_0)<c_1 \frac{R}{4\pi ^2 L} \right\} \right\} \\&= c_1 \frac{R}{\pi ^2 L}. \end{aligned}$$

This completes the proof of items $(i)-(ii)$. In order to prove item (*iii*), note that if $L \ge 25$, Then the connected components of *G* are larger than $\frac{2}{L^{1/2}}$, i.e. cover the circle twice. If in addition we require than $\sigma > \frac{2}{R}+ c_1 \frac{R}{\pi ^2\,L}$, then we can prove, using the techniques developed in section 6, that Hypotheses (H5*)–(H6*) are satisfied, and the claim follows from Remark 2.7$\square$

## Data Availability

Data sharing not applicable to this article as no datasets were generated or analysed during the current study.

## Footnotes

1 The precise value of $\delta _1$ is determined in Sect. 5.
